# Information Loss in Scalar Monetary Aggregation: A Tensorial Langevin Framework for Financial Shock Propagation and Policy Targeting

**DOI:** 10.3390/e28080915

**Published:** 2026-08-14

**Authors:** M. Rodrigo Pinheiro, Mario J. Pinheiro

**Affiliations:** 1 Vortex Legacy, 2800-260 Almada, Portugal; mp.pinheiromario@gmail.com; 2 Department of Physics, Instituto Superior Técnico (IST), Universidade de Lisboa, Av. Rovisco Pais 1, 1049-001 Lisboa, Portugal

**Keywords:** econophysics, information theory, entropy, coarse-graining, non-equilibrium dynamics, tensor representation, Lyapunov stability, financial contagion, policy optimization

## Abstract

We develop a tensor-based dynamical framework for monetary flows in multi-sector, multi-agent economies and quantify the information destroyed when the monetary state is reduced to a scalar aggregate. The state is a third-order tensor encoding capital flows across sectors, agent classes, and time; deviations from equilibrium obey a tensor-indexed Langevin (multivariate Ornstein–Uhlenbeck) equation with a coupling operator and channel-specific friction rates. Using standard Lyapunov theory, we assemble a stability and convergence framework for the induced vectorized system, with a bound stated so as to remain valid for the non-normal system matrices generated by asymmetric economic coupling, and characterize the stochastically forced case in the mean-square sense. Shannon entropy, Kullback–Leibler divergence, and sector–agent mutual information measure the structural information discarded by scalar aggregation. We then study a stylized, heuristically calibrated 3×3 economy subject to a shock inspired by the 2007–2009 crisis; we emphasize at the outset that the figures reported below are properties of that calibration and are not empirical estimates. In this scenario Finance absorbs an 18.9% peak capital loss while Manufacturing and Services suffer 5.8% and 3.9% secondary drops, against an aggregate contraction of only 8.6%; the Kullback–Leibler divergence of the sector–agent flow distribution recovers systematically later than the aggregate signal, a lag that is positive in 96.6% of a 1000-draw Monte Carlo ensemble, although its magnitude is calibration-dependent. Under a symmetric exit rule, a deficit-targeted stimulus restores equilibrium substantially faster than a share-weighted uniform stimulus in 100% of the ensemble while spending strictly less—its realized expenditure saturates below the uniform budget because it self-terminates as deficits close—and attains integrated disequilibrium within 18% of the exact linear-quadratic optimum at equal control effort while requiring no knowledge of the system matrix. The ordinal conclusions—aggregation masks the epicenter, structure lags the aggregate, and deficit targeting dominates uniformity—are robust across a wide neighborhood of the calibration, and identify the disaggregated state as the object that stabilization policy needs and that scalar aggregation destroys.

## 1. Introduction

Classical monetary theory represents money as a scalar quantity: a unidimensional measure of value that reduces economic activity to additive, linearly separable transactions [1]. While this abstraction suffices for aggregate accounting, it obscures the heterogeneous, non-equilibrium structure of real financial systems. The 2008 global financial crisis illustrated this failure concisely: aggregate credit statistics signaled only a modest decline in total lending, while the sector-specific collapse of credit to manufacturing and household consumption—transmitted through the banking system—drove cascading real-economy contractions invisible to scalar metrics [2].

From the perspective of complex-systems theory, an economy is a high-dimensional nonlinear dynamical system operating far from equilibrium [3], a view developed in detail by the heterogeneous-agent and agent-based macroeconomics literature [4,5]. Its state cannot be faithfully projected onto a scalar without information loss. The mathematical language appropriate to this description is not scalar algebra but tensor analysis: the same language that physics uses to represent quantities whose structure depends simultaneously on multiple independent dimensions (e.g., the stress tensor in continuum mechanics, or the conductivity tensor in anisotropic materials) [6,7,8]. Crucially, the statement “scalar aggregation loses information” can be made quantitative with the standard tools of information theory [9,10]: entropy, Kullback–Leibler (KL) divergence, and mutual information.

This article formalizes that intuition. We are explicit about what in what follows is standard and what is not. The dynamical skeleton—a linear dissipative system with additive forcing—is a multivariate Ornstein–Uhlenbeck process, and Proposition 1 is a textbook result in the theory of continuous-time Lyapunov equations, reproduced here only to fix notation and because the bound (22) is used quantitatively in Section 7. Neither is offered as a theoretical contribution.

The contribution of the paper is the combination of that standard skeleton with three elements that are not available to a scalar or unstructured-vector formulation:

**(a) A quantitative account of aggregation loss.** We treat scalar aggregation as a coarse-graining map and measure the discarded information by the Shannon entropy of the normalized flow distribution, its Kullback–Leibler divergence from equilibrium, and the sector–agent mutual information. These are functions of the index structure and are undefined for an unstructured state vector (Section 4.3). We show that the KL divergence recovers systematically later than the aggregate signal, so that the two observables disagree about when a crisis has ended.

**(b) A demonstration that the discarded component is the policy-relevant one.** We compare a stimulus rule conditioned on the disaggregated state against one conditioned only on aggregate information, under equal budgets and a common termination rule, and benchmark both against the exact linear-quadratic optimum. The targeted rule captures most of the attainable optimal-control gain without knowledge of the system matrix, which locates the value in the *observation* of the disaggregated state rather than in the solution of the control problem.

**(c) A proof-of-concept framework with a specified empirical route.** The three-sector economy of Section 7 is illustrative, and we say so throughout; Section 3 specifies the calibration protocol—sectoral partition from input–output accounts, agent classes from the institutional-sector breakdown of the national accounts, coupling estimated from credit registers using tensor factor methods—by which the framework would be taken to data.

We regard (a) and (b) as the substantive claims and (c) as a programme. What the paper does *not* establish is equally worth stating: it does not estimate the coupling operator from data, it does not test the framework empirically, and its policy conclusions hold within the class of linear, single-equilibrium models studied here (Section 10).

The remainder is structured as follows. Section 2 surveys the literature on which the framework draws and states its positioning against them. Section 3 sets out the methodology and numerical design. Section 4 develops the tensor representation and its dynamical equations, and motivates the tensor formulation against its vectorization. Section 5 formalizes scalar aggregation as coarse-graining and introduces the entropic diagnostics. Section 6 provides the Lyapunov stability analysis. Section 7 introduces the toy model and numerical results, including the information-theoretic diagnostics. Section 8 translates the theory into policy-design principles, tests their robustness, and benchmarks them against exact optimal control. Section 9 discusses the results and their positioning in the literature. Section 10 addresses limitations and ethical considerations. Section 11 concludes with theoretical and practical implications.

## 2. Related Work and Theoretical Background

The framework developed below draws on five bodies of literature. We summarize each, and state in Section 2.7 what the present paper takes from it and what it adds.

### 2.1. Financial Contagion in Networks

The modern theory of financial contagion models the propagation of distress across a network of balance-sheet exposures. Allen and Gale [11] established that the completeness of the interbank network determines whether idiosyncratic shocks are dissipated or amplified. Gai and Kapadia [12] characterized the resulting cascades as a percolation phenomenon, with the now-standard finding that contagion is a low-probability/high-impact event whose likelihood is non-monotone in connectivity. Battiston and co-authors [13,14] introduced DebtRank as a recursive measure of systemic impact and showed that increasing connectivity produces a trade-off between risk-sharing and systemic risk. Elliott, Golub and Jackson [15] decomposed this into integration and diversification, and Acemoglu, Ozdaglar and Tahbaz-Salehi [16] established the robust-yet-fragile property: dense networks absorb small shocks and amplify large ones. Gualdi and co-authors [17] address the reconstruction of the relevant network from partial data, which is the estimation problem any applied version of the present framework must solve.

The relation to our formulation is direct. The coupling operator K of Equation (10) plays, in linear-response form, the role that the exposure network plays in this literature: its off-diagonal entries are the channels along which distress travels, and its spectral properties determine whether distress is absorbed or amplified, exactly as the spectral condition α(A)<0 of Proposition 1 makes precise. Two differences matter. First, the state here is indexed by sector *and* agent class rather than by institution, which is what makes the sector–agent mutual information of Equation (18) definable and is the source of the diagnostics of Section 5. Second, this literature is concerned predominantly with the *occurrence* of cascades, whereas the question posed here is what an observer who sees only the aggregate can infer about a cascade that has already occurred.

### 2.2. International Transmission of Financial Shocks

A parallel literature studies transmission across borders. Kaminsky and Reinhart [18] identified trade and common creditors as the dominant channels; Forbes and Rigobon [19] drew the distinction, essential for the interpretation of any coupling estimate, between contagion—a shift in the propagation mechanism during crisis—and mere interdependence, a stable comovement that heteroskedasticity can make look like contagion. Cetorelli and Goldberg [20] documented the global-bank channel during 2007–2009, and Kalemli-Özcan, Papaioannou and Perri [21] showed that financial integration reduces business-cycle synchronization in normal times and raises it during crises—a state dependence that our time-invariant K does not capture and that we flag as a limitation in Section 10. Rey [22] established the global financial cycle as a common factor constraining national policy independence.

This literature bears on the present work in two ways. It supplies the empirical warrant for treating the coupling entries as crisis-specific rather than structural, and it identifies the natural extension of the framework: a cross-country version corresponds to adding a fourth mode to the state tensor, with K acquiring a country-pair block structure. We note this as a specific direction in Section 11 rather than as a generality.

### 2.3. Production Networks and the Granular Origins of Aggregate Fluctuations

Gabaix [23] showed that idiosyncratic shocks to large firms do not wash out in aggregate when the firm-size distribution is fat-tailed; Acemoglu and co-authors [2] established the analogous result for input–output linkages, with aggregate volatility governed by the network’s degree sequence rather than by $1/\sqrt{n}$ averaging. Carvalho [24] surveys the micro-to-macro transmission this implies, and Baqaee and Farhi [25] extend it beyond the first-order (Hulten) approximation to show that network structure matters at second order even when it does not at first.

This is the empirical and theoretical background for the coupling structure specified in Section 7, and we use it there to justify the sign pattern of the coupling entries rather than presenting them as free stipulations. It is also the literature that most directly supports the paper’s motivating premise: if aggregate fluctuations have disaggregated origins, then an aggregate observable is a lossy summary of the object that generates them.

### 2.4. Entropy and Information Theory in Economics and Finance

The application of information-theoretic functionals to economic distributions is long established. Theil [26] introduced entropy measures of concentration and inequality into economics, and Jaynes [27] supplied the maximum-entropy framework on which much subsequent work rests. In finance, Chakraborti and co-authors [28] survey the econophysics use of entropy-based measures, and Zhou, Cai and Tong [29] review applications ranging from portfolio selection to risk measurement.

We state plainly that applying entropy and KL divergence to economic coarse-graining has precedent, and that the present paper does not claim that application as novel. What we have not found in this literature is the specific construction used here: the KL divergence of the *normalized sector–agent flow distribution relative to its own equilibrium* used as a real-time crisis diagnostic, with its recovery time compared directly against that of the corresponding aggregate. It is the comparison of recovery times, rather than the functional, that carries the argument of Section 7.4.

### 2.5. Tensor Methods in Economics and Econometrics

Kolda and Bader [7] is the standard reference for tensor decompositions; Cichocki and co-authors [30] survey low-rank and tensor-network formats for large-scale problems. In econometrics, Chen, Yang, and Zhang [31] develop factor models for high-dimensional tensor time series; Han, Chen, and Zhang [32] address rank determination, and Babii, Ghysels and Pan [8] develop tensor principal component analysis for economic data.

This literature defines the state of the art for the estimation problem that the present framework would face if applied to data, and we rely on it in two places: the parsimony argument of Section 4.3(ii), which is a claim about *estimability* conditional on low-rank structure and not a claim that we have established such structure (see Section 10), and the calibration protocol of Section 3.8.

### 2.6. Optimal Policy Under Imperfect Information

The policy comparison of Section 8 is an instance of a general problem: policy conditioned on a coarse or noisy observation of the state. Woodford [33] develops the theory of optimal monetary policy under a specified information structure, and Svensson and Woodford [34] characterize the indicator variables on which an optimal rule should condition. Orphanides [35] shows that policy evaluated with real-time rather than revised data can reverse the assessment of a rule’s performance, and Bernanke [36] reviews the declining reliability of monetary aggregates as policy indicators—a judgement made on empirical grounds that the present framework recovers from an information-theoretic one.

### 2.7. Positioning of the Present Paper

The framework takes its propagation structure from the contagion and production-network literatures, its diagnostics from information theory, its estimation strategy from tensor econometrics, and its policy question from the imperfect-information literature. What it adds is a single joint treatment: the same object—the sector–agent flow tensor—serves as the state whose dynamics propagate the shock, as the fine-grained object whose coarse-graining is measured, and as the domain on which policy allocates. The claim that scalar aggregation is lossy is available in each of these literatures separately; what is new here is a measurement of that loss in information-theoretic units, together with a demonstration, against an exact optimal-control frontier, that the lost component is the one that determines policy efficacy.

## 3. Methodology and Numerical Design

This is a theoretical and computational study. There is no empirical sample: the data analyzed are generated by simulation from a stipulated model, and every quantitative statement in the paper is a property of that model under a specified calibration. This section sets out the objects of study, the parameter specification and its status, the closed-loop systems that are actually integrated, the numerical scheme, the operational definition of every reported variable, the design of the Monte Carlo experiment, and the protocol by which the framework would be calibrated to data.

### 3.1. Objects of Study

The unit of analysis is the *sector–agent channel* (i,j), $i=1,\ldots ,N_{S}$, $j=1,\ldots ,N_{A}$. The toy economy of Section 7 has $N_{S}=N_{A}=3$, hence nine channels. The simulated object is the deviation trajectory $x\left(t\right)\in R^{N_{S}N_{A}}$ together with the functionals of it defined in Section 3.6.

### 3.2. Parameter Specification and Its Status

The equilibrium matrix $M^{*}$ (24), the friction rates Γ (25), the eleven coupling entries specified in Section 7 and the shock profile (31) are *heuristically specified* to represent the stylized transmission channels of 2007–2009. The magnitudes are chosen so that the resulting system is stable, the implied characteristic recovery times $\tau_{ij}=\Gamma_{ij}^{-1}$ lie in a plausible 6–20 quarter range, and the shock ordering reproduces the observed sequence in which financial distress preceded real-sector contraction. No parameter is estimated. We state this here, before any result is reported, because the register in which the results are presented depends on it: throughout the paper, peak drops, recovery times and the structural lag are illustrative properties of a stylized calibration, not empirical estimates. Section 8.4 assesses which conclusions survive perturbation of the calibration, and Section 10 states what that assessment does and does not establish.

### 3.3. Time Period and Horizon

Time is continuous and measured in quarters. The shock is applied at t=0; baseline figures are integrated to T=80 quarters and the Monte Carlo ensemble to T=120 quarters, the longer horizon being required because the non-terminating uniform policy has not recovered by 80 quarters in part of the ensemble. Policy is deployed at $t_{0}=8$ quarters, two quarters after the shock peak, and where a termination rule applies, the policy window is $W=[t_{0},\;t_{0}+T_{\mathrm{pol}}]$ with $T_{\mathrm{pol}}=8$ quarters. The correspondence to the 2007–2009 episode is interpretive: the simulation is not dated to calendar time and no quarter of the simulation should be read as a specific historical quarter.

### 3.4. Policy Rules as Closed-Loop Systems

A policy enters as the forcing $s^{\mathrm{pol}}\left(t\right)$ in Equation (32). The two heuristics differ in a respect that governs how the resulting system must be solved: the uniform rule is an *open-loop* forcing, whereas the targeted rule is a *state feedback*.

*Uniform (scalar-information) rule.* With equilibrium shares $w_{ij}=M_{ij}^{*}/\sum_{kl}M_{kl}^{*}$ and $1_{W}\left(t\right)$ the indicator of the policy window,

(1) $$s_{ij}^{\mathrm{uni}}\left(t\right)=\lambda \;w_{ij}\;1_{W}\left(t\right)\;(\mathrm{terminating}\;\mathrm{specification},\;\mathrm{primary}),$$

(2) $$s_{ij}^{\mathrm{uni}}\left(t\right)=\lambda \;w_{ij}\;e^{-0.06(t-t_{0})}\;1_{\left\{t\geq t_{0}\right\}}\;(\mathrm{non-terminating}\;\mathrm{specification},\;\mathrm{secondary}).$$

Since $s^{\mathrm{uni}}$ does not depend on x, the closed loop $\dot{x}=Ax+s^{\mathrm{shock}}\left(t\right)+s^{\mathrm{uni}}\left(t\right)$ remains linear and admits the variation-of-constants solution; we nonetheless integrate it numerically, with the same scheme used for the targeted rule, so that the two are compared under identical numerical treatment.

*Targeted (tensor-information) rule.* With channel deficits $d_{ij}\left(x\right)=\max\left(-x_{ij},\;0\right)$ and total deficit $D\left(x\right)=\sum_{kl}d_{kl}\left(x\right)$,

(3) $$s_{ij}^{\mathrm{tgt}}\left(t\right)=\lambda \;\frac{d_{ij}\left(x\left(t\right)\right)}{D(x(t))}\;1_{W}\left(t\right),\;s_{ij}^{\mathrm{tgt}}\left(t\right):=0\;\;\mathrm{when}\;D\left(x\left(t\right)\right)=0.$$

Because $s^{\mathrm{tgt}}$ depends on the current state, the closed loop

(4) $$\dot{x}=Ax+s^{\mathrm{shock}}\left(t\right)+s^{\mathrm{tgt}}\left(x\left(t\right),t\right)$$

is a *nonlinear, piecewise-smooth* ordinary differential equation: the map x↦d(x)/D(x) is continuous but not differentiable on the hyperplanes $x_{ij}=0$, and is homogeneous of degree zero, so that the total injection rate equals λ whenever any deficit remains and falls to zero once all deficits close. This last property is the self-termination discussed in Section 8.3, and it is the reason the targeted rule spends less than its nominal budget over a fixed window. Section 8.2 shows that this imbalance cannot be removed by raising $\lambda^{\mathrm{tgt}}$: realized expenditure of the targeted rule saturates strictly below the uniform rule’s budget, so the comparison is conservative in the targeted rule’s favour.

*Optimal benchmark.* The LQR feedback of Section 8.1 is likewise a state feedback, but a linear one, so the closed loop $\dot{x}=\left(A-\rho^{-1}BB^{⊤}S\right)x+s^{\mathrm{shock}}\left(t\right)$ is linear and time-invariant after $t_{0}$.

### 3.5. Numerical Scheme and Verification

All systems are integrated from x(0)=0 by the explicit Runge–Kutta method of Dormand and Prince [37] as implemented in scipy.integrate.solve_ivp [38] with method=“RK45”, rtol $=10^{-8}$ and atol $=10^{-10}$, on a uniform output grid of 0.05 q.

The closed-loop system (4) under the targeted rule is only piecewise smooth, and this has a practical consequence worth recording. As a channel crosses $x_{ij}=0$ the allocation direction d(x)/D(x) flips discontinuously; adaptive error control responds by driving the step size down, so that neither an adaptive explicit scheme at tight tolerance nor an implicit stiff scheme is a practical instrument here—the latter additionally estimates a Jacobian that does not exist at the kink. We therefore verify the policy trajectories with a *fixed-step* classical Runge–Kutta scheme, which cannot chatter, and check self-convergence by halving the step: between Δt=0.002 and Δt=0.001 q the maximum discrepancy in V(t) over the horizon is $3.0\times 10^{-4}$, i.e., $5.5\times 10^{-5}$ relative to $\max_{t}V$, and the recovery time is unchanged at $\tau_{r}=12.81$ q. As a second and independent check, we replace the kink by the smooth surrogate $\varepsilon \log(1+e^{-x/\varepsilon })$ and let ε→0; the recovery time converges monotonically (12.39, 12.22, 12.20 q for $\varepsilon =10^{-2},10^{-3},10^{-4}$), confirming that the reported values are properties of the model and not of the regularization. The algebraic Lyapunov and Riccati equations are solved by Schur methods [39] (scipy.linalg.solve_lyapunov and scipy.linalg.solve_continuous_are); residual norms are reported in the Supplementary Material.

### 3.6. Measurement of Reported Variables

Table 1 defines every outcome measure used in the paper, with its symbol, operational definition, units, and the figure or table in which it is reported. Two definitions require comment. The recovery time $\tau_{r}$ is the first grid time at which V(t) falls below 5% of its trajectory maximum *and remains below it thereafter*; the persistence clause matters because the uniform-policy trajectories are non-monotone. The reallocation share δ(t) is the total variation distance between the current and equilibrium flow distributions, and is included because it converts the KL divergence into an economically interpretable quantity: δ is the fraction of aggregate capital flow that would have to be moved between channels to restore the equilibrium composition (Section 5).

### 3.7. Design of the Monte Carlo Experiment

The sampling design of Section 8.4 is as follows. N=1000 economies are drawn; in each, every non-zero coupling entry is independently rescaled by a factor ∼U[0.5,1.5], every friction rate by U[0.7,1.3], every shock amplitude by U[0.7,1.3] and every decay rate by U[0.8,1.2]. Draws are screened for stability (α(A)<0) and for strict positivity of all flows, both of which hold for all 1000 draws; had a draw failed either screen, it would have been reported rather than discarded, and we state the screen here so that the absence of rejections is verifiable. Each draw is integrated under five policy scenarios over 120 quarters, and recovery times are right-censored at that horizon, with the censoring rate reported in Section 8.4. Random seeds are fixed and recorded in the public repository.

### 3.8. Protocol for Empirical Calibration

Although no estimation is performed here, we specify the design of the empirical study the framework points to, since the value of the proof-of-concept depends on that route being concrete. The sectoral partition would be taken from national input–output accounts (the OECD ICIO or WIOD tables); agent classes from the institutional-sector breakdown of the national accounts (households and NPISH, non-financial corporations, financial corporations, general government); and the coupling operator estimated from supervisory credit-register data—AnaCredit at the euro-area level, or the Central de Responsabilidades de Crédito for Portugal—at quarterly frequency. Identification of K from such data is a high-dimensional problem in which the low-rank tensor factor methods of Chen, Yang and Zhang [31] and the rank-selection procedure of Han, Chen and Zhang [32] are the natural tools; whether the estimated operator is in fact well approximated at low rank is an empirical question, not an assumption we are entitled to make in advance (Section 10). The privacy and governance constraints on access to such data are discussed in Section 10.2.

## 4. The Monetary Tensor: Framework and Dynamics

### 4.1. Representation

Let an economy consist of $N_{S}$ productive sectors (e.g., manufacturing, finance, services) and $N_{A}$ agent classes (e.g., households, firms, government). The *monetary tensor* $M\in R^{N_{S}\times N_{A}\times T}$ is defined such that $M_{ijk}$ represents the capital flow directed from sector *i* toward agent class *j* during period *k*.

The underlying process is continuous in time; the tensor M collects its values on a quarterly sampling grid ${\left\{t_{k}\right\}}_{k=1}^{T}$. The *monetary matrix* $M\left(t\right)\in R^{N_{S}\times N_{A}}$ is the corresponding mode-3 slice,

(5) $${\left[M(t_{k})\right]}_{ij}\;:=\;M_{ijk},$$

extended to continuous *t* by the dynamics of Equation (10). No marginalization over the temporal index is involved.

A scalar monetary model retains only a single functional of M(t), such as the total $S\left(t\right)=\sum_{ij}M_{ij}\left(t\right)$ or the Frobenius norm ${∥M\left(t\right)∥}_{F}={\left(\sum_{ij}M_{ij}^{2}\right)}^{1/2}$, discarding all directional and sectoral information.

Every tensor admits a CP decomposition [7] for *R* sufficiently large, so the decomposition carries content only when *R* is small relative to the ambient dimensions. We therefore consider throughout the regime in which M admits an accurate rank-*R* approximation with $R\ll \min(N_{S},N_{A},T)$, and we treat this as a hypothesis to be tested at the calibration stage [32] rather than as a property established here:

(6) $$M=\sum_{r=1}^{R}\lambda_{r}\;s^{\left(r\right)}⊗a^{\left(r\right)}⊗t^{\left(r\right)},$$

where $s^{\left(r\right)}\in R^{N_{S}}$, $a^{\left(r\right)}\in R^{N_{A}}$, $t^{\left(r\right)}\in R^{T}$ are, respectively, sectoral, agent-type, and temporal factor vectors, and $\lambda_{r}>0$ are mode amplitudes. The rank-1 approximation (R=1)—used in earlier work as a simplification—corresponds to separating sectoral, agent, and temporal effects:

(7) $$M\left(t\right)\approx \lambda_{1}\;s^{\left(1\right)}⊗a^{\left(1\right)}.$$

We make no such assumption in what follows; the full matrix M(t) evolves according to coupled dynamics in the deviation variable, in a linear-response regime around equilibrium (see Section 10 for the limitations of this regime).

### 4.2. Governing Equation

Let $x_{ij}\left(t\right)=M_{ij}\left(t\right)-M_{ij}^{*}$ denote the deviation of the monetary flow from its equilibrium value $M_{ij}^{*}>0$.

The coupling object is a fourth-order tensor $K\in R^{N_{S}\times N_{A}\times N_{S}\times N_{A}}$ acting on the matrix slice $x\left(t\right)\in R^{N_{S}\times N_{A}}$ by contraction over the last two indices,

(8) $${\left(K\;\cdot \;x\right)}_{ij}\;=\;\sum_{k,l}K_{ijkl}\;x_{kl},$$

with *no* temporal coupling: the third mode of M indexes time, which is the evolution parameter rather than a contracted index, so the dynamics are local in time and no convolution is involved. Two conventions follow and are used throughout. First, the index order of K is *target-first*: in $K_{\left(ij)(kl\right)}$ the pair (ij) is the channel affected and (kl) the channel from which the disturbance originates, since Equation (10) places $\sum_{kl}K_{ijkl}x_{kl}$ in the equation for ${\dot{x}}_{ij}$; equivalently, an entry of K is read as source (kl)→ target (ij). Second, friction is a property of the channel and not of time,

(9) $$\Gamma_{ijk}=\Gamma_{ij}\;\mathrm{for}\;\mathrm{all}\;k,$$

an assumption we make explicit here because it is what permits the flattening below; a genuinely time-dependent Γ(t) would produce a time-varying system matrix and place the analysis outside Proposition 1 (see Section 10).

We propose the following *tensorial Langevin equation*:

(10) $$\frac{dx_{ij}}{dt}=-\Gamma_{ij}\;x_{ij}+\sum_{k,l}K_{ijkl}\;x_{kl}+s_{ij}\left(t\right)+\sigma_{ij}\;\xi_{ij}\left(t\right),$$

where:

- $\Gamma_{ij}>0$ is the *sector–agent friction rate*, encoding mean-reversion speed (inverse of characteristic recovery time);
- $K_{ijkl}$ is the *coupling tensor*, representing how deviations in sector-*k*/agent-*l* flows propagate to sector-*i*/agent-*j* (credit-channel, supply-chain, or demand-side spillovers);
- $s_{ij}\left(t\right)$ is a deterministic external forcing (shocks, policy);
- $\xi_{ij}\left(t\right)$ is white noise with amplitude $\sigma_{ij}$.

We use the term *Langevin* in the sense customary in econophysics—a linear dissipative system with additive stochastic forcing, i.e., a multivariate Ornstein–Uhlenbeck process. We interpret Equation (10) in the Itô sense throughout; for a linear system with additive (state-independent) noise, the Itô and Stratonovich interpretations coincide, so this choice affects neither the stationary covariance of Remark 2 nor the Euler–Maruyama simulation of Section 7.5, which is the Itô discretization. We impose no fluctuation–dissipation relation between $D=\mathrm{diag}(\sigma_{ij}^{2})$ and the dissipative part of the dynamics, and no such relation is claimed or used anywhere below; there is no temperature-like parameter in the model that would make one meaningful.

Equations (8) and (9) make the vectorization explicit. Fix the index map $a\left(i,j\right)=\left(i-1\right)N_{A}+j$ and let x(t)=vec(x) with $x_{a(i,j)}=x_{ij}$. Defining

(11) $${\left(K_{\mathrm{flat}}\right)}_{a(i,j),\;b(k,l)}=K_{ijkl},\;\Gamma =\mathrm{vec}\left(\Gamma \right),$$

we have ${\left(K\;\cdot \;x\right)}_{ij}={\left(K_{\mathrm{flat}}x\right)}_{a(i,j)}$ identically, and the friction term becomes diag(Γ)x. The system matrix is therefore

(12) $$A=K_{\mathrm{flat}}-\mathrm{diag}\left(\Gamma \right),$$

and the deterministic part of Equation (10) becomes

(13) $$\dot{x}=A\;x+s\left(t\right)$$

with no further approximation. Note that diag(Γ), which enters *A*, is an $N_{S}N_{A}\times N_{S}N_{A}$ diagonal matrix, whereas the array Γ displayed in Equation (25) collects the channel-wise rates $\Gamma_{ij}$; the two objects are distinct and are named separately throughout.

*Economic interpretation of A.* The diagonal entries $-\Gamma_{ij}$ model channel-level mean-reversion (the tendency of credit markets to self-correct). The off-diagonal entries $K_{ijkl}$ encode inter-sectoral propagation; negative entries represent *contagion* (a collapse in Finance propagates negatively to Manufacturing credit), while positive entries represent *complementarity* (Manufacturing activity sustains Service-sector demand). Because credit-channel couplings are generically asymmetric ($K_{ijkl}\neq K_{klij}$), the system matrix *A* is in general *non-normal*; the dynamical consequences of this fact are made precise in Section 6.

### 4.3. Why a Tensor and Not a Vector?

Since Equation (10) vectorizes exactly into the linear system (13), it is legitimate to ask what the tensor formulation adds beyond notation: for the dynamics of the 3×3 toy model of Section 7, the vectorized representation is dynamically equivalent, and we make no claim to the contrary. The tensor structure is essential at three other levels of the analysis.

*(i) The information-theoretic diagnostics are functions of the index structure, not of the vector.* The mutual information I(S;A) of Equation (18) requires the marginals $p_{i\cdot }$ and $p_{\cdot j}$, which exist only because the state carries a sector×agent factorization; for an unstructured vector in $R^{N_{S}N_{A}}$, I(S;A) is not even defined. The same holds for the statement that $M^{*}$ is not rank-1 (Section 7.4): matrix rank, and with it the information-theoretic insufficiency of the separable approximation (7), is invisible after vectorization. The central diagnostics of this paper—which sector–agent channels are displaced, and whether sectoral origin and agent destination have become statistically coupled during a crisis—are therefore tensorial observables in an essential sense.

*(ii) Parametric parsimony at scale.* A generic coupling operator on the vectorized state has ${\left(N_{S}N_{A}\right)}^{2}$ free parameters, which is hopeless to estimate for realistic dimensions (e.g., $N_{S}=50$ sectors and $N_{A}=10$ agent classes give $2.5\times 10^{5}$ parameters). The tensor structure *makes available* low-rank parameterizations of K—CP or Tucker formats, or sums of Kronecker products [7,8,30]—which reduce the parameter count to $O(R\left(N_{S}+N_{A}\right))$ and would make empirical calibration from input–output tables and credit registers (Section 3.8) statistically feasible. We stress that this advantage is *conditional* on the coupling operator being well approximated at low rank, which is an empirical question we do not settle here; the Supplementary Material reports the CP-fit residual of the simulated trajectory tensor as a function of *R*: the relative residual $∥M-\hat{M}{∥}_{F}/{∥M∥}_{F}$ falls from $2.7\times 10^{-1}$ at R=1 to $3.5\times 10^{-2}$ at R=2 and $7.4\times 10^{-3}$ at R=3. We draw no conclusion from this. A 3×3×T tensor has CP rank at most three, so the exercise cannot discriminate between genuine low-rank structure and the exhaustion of the available rank, and it says nothing about the $N_{S}=50$ case that motivates the argument.

*(iii) Policy lives on the indices.* The targeted rule of Section 8 allocates resources to cells (i,j), and its transmission-channel interpretation (Finance–Firms credit, Manufacturing–Household demand) is a statement about index pairs. More fundamentally, the informational asymmetry that drives the policy results—a scalar authority observes $S\left(t\right)=\sum_{ij}M_{ij}\left(t\right)$, a tensor authority observes the full array $M_{ij}\left(t\right)$—can only be formulated once the state is indexed. The paper’s central claim, that scalar aggregation is a lossy coarse-graining map, presupposes the tensor as the fine-grained object being coarse-grained.

In short, the tensor formulation is not needed to *integrate* the toy model, but it is needed to *diagnose* it (i), to *scale* it (ii), and to *act* on it (iii). We regard the dynamical equation itself—a multivariate Ornstein–Uhlenbeck system—as standard, and the contribution of this paper as the combination of that standard dynamical skeleton with the tensorial coarse-graining analysis, the entropic crisis diagnostics, and the policy-targeting results built on them.

### 4.4. Notation

Table 2 summarizes the notation used throughout the paper and in all figure captions.

## 5. Coarse-Graining, Entropy, and Information Loss

Scalar monetary aggregation is a coarse-graining operation: the map $M\left(t\right)\mapsto S\left(t\right)\equiv \sum_{ij}M_{ij}\left(t\right)$ retains a single degree of freedom out of $N_{S}N_{A}$, discarding the entire *distributional structure* of capital flows. This section quantifies the discarded information [9,10].

The functional S(t) is the total gross capital flow across all sector–agent channels in a period, and is the model analog of an aggregate credit or gross-lending statistic. We adopt it because it is the aggregate whose real-world counterpart is the object of the motivating observation of Section 1: during 2007–2009 aggregate lending statistics recorded a modest decline while sectoral credit collapsed. The choice is a modeling decision rather than a derivation, and other scalarizations—the Frobenius norm ${∥M\left(t\right)∥}_{F}$, the leading singular value $\sigma_{1}\left(M\left(t\right)\right)$, or a size-weighted sum—would produce different aggregates and, in principle, different degrees of information loss. We report the trajectories of ${∥M\left(t\right)∥}_{F}$ and $\sigma_{1}\left(M\left(t\right)\right)$ alongside S(t) in the Supplementary Material. In the stylized calibration all three understate the epicenter: against a Finance-sector peak drop of 18.9%, the sum contracts by 8.6%, the Frobenius norm by 10.1% and the leading singular value by 8.6%, with recovery times of 17.0, 19.2 and 16.2 quarters respectively. The conclusion of Section 7 is therefore not an artefact of the particular functional chosen. We note that neither ${∥\cdot ∥}_{F}$ nor $\sigma_{1}$ is additive across channels, so neither corresponds to any published aggregate statistic, which is a further reason to take the sum as the object whose informational deficiency is at issue.

Since all equilibrium flows satisfy $M_{ij}^{*}>0$ and flows remain strictly positive in the regime studied here (verified numerically in Section 7), the normalized flow distribution

(14) $$\;p_{ij}\left(t\right)=\frac{M_{ij}\left(t\right)}{\sum_{kl}M_{kl}\left(t\right)}$$

is well defined. Its Shannon entropy,

(15) $$H\left(t\right)=-\sum_{ij}p_{ij}\left(t\right)\;\ln p_{ij}\left(t\right),$$

measures the dispersion of capital across sector–agent channels, while the Kullback–Leibler divergence with respect to the equilibrium distribution $p_{ij}^{*}=M_{ij}^{*}/\sum_{kl}M_{kl}^{*}$,

(16) $$\;D_{\mathrm{KL}}\left(p\left(t\right)\;∥\;p^{*}\right)=\sum_{ij}p_{ij}\left(t\right)\;\ln\;\frac{p_{ij}\left(t\right)}{p_{ij}^{*}}\;\geq \;0,$$

measures the *structural* displacement of the economy from equilibrium, independently of the aggregate level S(t).

A divergence in nats is not by itself economically interpretable, and we therefore report alongside $D_{\mathrm{KL}}$ the total variation distance

(17) $$\;\delta \left(t\right)=\frac{1}{2}\sum_{ij}\left|p_{ij}\left(t\right)-p_{ij}^{*}\right|,$$

which admits a direct reading: δ(t) is the fraction of aggregate capital flow that would have to be reallocated between sector–agent channels to restore the equilibrium composition. Pinsker’s inequality [10] (Lemma 11.6.1) gives $\delta \leq \sqrt{D_{\mathrm{KL}}/2}$, so the peak divergence of $8.5\times 10^{-3}$ nats reported in Section 7.4 corresponds to a reallocation share of at most 6.5%; the realized value is $\delta_{\max}=5.2\%$, so Pinsker’s bound is loose here by a factor of 1.27. The economic reading is that at the trough of the crisis, and in this calibration, about one twentieth of aggregate capital flow sits in a different sector–agent channel from the one it would occupy at equilibrium.

Two reference points fix the scale. First, a *purely aggregate* shock—one that scales every channel by a common factor—leaves $p_{ij}$ unchanged and yields $D_{\mathrm{KL}}\equiv 0$ identically. This is the appropriate null: it establishes that the entire measured divergence is compositional, and that a scalar observer would by construction see none of it, however large the aggregate movement. We verify this numerically: applying a proportional shock of the same aggregate magnitude as the simulated one gives $\max_{t}D_{\mathrm{KL}}=3.2\times 10^{-16}$ nats and $\max_{t}\delta =1.6\times 10^{-14}\%$, i.e., zero to machine precision. The entire divergence measured in Section 7.4 is therefore compositional, and a scalar observer sees none of it however large the aggregate movement.

The two observables S(t) and $D_{\mathrm{KL}}\left(t\right)$ are informationally complementary: an economy may exhibit $S\left(t\right)\approx S^{*}$ (no aggregate anomaly) while $D_{\mathrm{KL}}\left(t\right)\gg 0$ (severe sectoral misallocation). This is precisely the regime realized in the stylized scenario of Section 7: in that calibration the aggregate recovers to within 5% of its peak anomaly after 17 quarters, whereas the structural displacement $D_{\mathrm{KL}}\left(t\right)$ takes 38 quarters—a lag during which the scalar signal reports near-normality while the sectoral composition of flows remains significantly distorted. As Section 8.4 shows, the sign of this lag is robust across calibrations while its magnitude is not.

Finally, treating $p_{ij}$ as a joint distribution over the sector variable *S* and the agent variable *A*, the mutual information

(18) $$I\left(S;A\right)\left(t\right)=\sum_{ij}p_{ij}\left(t\right)\;\ln\;\frac{p_{ij}\left(t\right)}{p_{i\cdot }\left(t\right)\;p_{\cdot j}\left(t\right)},$$

with marginals $p_{i\cdot }=\sum_{j}p_{ij}$ and $p_{\cdot j}=\sum_{i}p_{ij}$, quantifies the statistical dependence between sectoral origin and agent destination of capital flows. A nonzero I(S;A) certifies that the flow matrix is not of rank one, i.e., that the separable approximation (7) is information-theoretically insufficient; the coupling $K_{ijkl}$ generates precisely such sector–agent correlations during crises.

In this formulation, the central claim of the paper acquires an information-theoretic statement: *scalar aggregation destroys the component of the monetary state—the distribution $p_{ij}$—that governs both shock propagation and the efficacy of stabilization policy*. The targeted stimulus of Section 8 is effective exactly because it conditions on the disaggregated state; the uniform stimulus underperforms because it conditions only on information available at the aggregate level.

## 6. Stability Analysis

### 6.1. Lyapunov Function

The stability of the zero equilibrium $x^{*}=0$ of the unforced system $\dot{x}=Ax$ is characterized by the following result, which is standard in the theory of continuous-time Lyapunov equations [40] (§2.2, Theorem 2.2.3, pp. 95–98), [41] (Theorem 4.6, pp. 136–137); we state it as a proposition, and include the short proof only to fix notation and because the bound (22) is used quantitatively in Section 7.

**Proposition** **1** (Global asymptotic stability)**.**
*Suppose the spectral abscissa of A satisfies α(A)≡maxiReλi(A)<0. Then there exists a unique symmetric positive-definite matrix $P\in R^{\left(N_{S}N_{A}\right)\times \left(N_{S}N_{A}\right)}$ satisfying the continuous-time Lyapunov equation*

(19) $$A^{⊤}P+PA=-I_{N_{S}N_{A}},$$

*and the function $V\left(x\right)=x^{⊤}P\;x$ is a global Lyapunov function for the system, satisfying*

(20) $$\dot{V}\left(t\right)=-{∥x\left(t\right)∥}^{2}\leq 0,$$

*with equality only at x=0. Moreover,*

(21) $$V\left(t\right)\;\leq \;V\left(t_{0}\right)\;e^{-\left(t-t_{0}\right)/\lambda_{\max}\left(P\right)},$$

*and consequently*

(22) $$∥x\left(t\right)∥\;\leq \;\sqrt{\kappa (P)}\;∥x\left(t_{0}\right)∥\;e^{-\left(t-t_{0}\right)/2\lambda_{\max}\left(P\right)},$$

*where $\kappa \left(P\right)=\lambda_{\max}\left(P\right)/\lambda_{\min}\left(P\right)$ is the condition number of P.*

**Proof.**  Existence and uniqueness of P≻0 follow from the standard theory of continuous-time Lyapunov equations, valid precisely when α(A)<0 [40] (§2.2, pp. 95–98). Differentiating V along trajectories [41] (§4.1, pp. 111–116),

(23) $$\dot{V}={\dot{x}}^{⊤}Px+x^{⊤}P\dot{x}=x^{⊤}\left(A^{⊤}P+PA\right)x=-{∥x∥}^{2}.$$

Since $V\leq \lambda_{\max}\left(P\right)\;{∥x∥}^{2}$, we obtain $\dot{V}\leq -V/\lambda_{\max}\left(P\right)$, and  Grönwall’s inequality yields (21). Bound (22) then follows from $\lambda_{\min}{\left(P\right)∥x∥}^{2}\leq V\leq \lambda_{\max}\left(P\right){∥x∥}^{2}$.    □

**Remark** **1** (Asymptotic rate and non-normality)**.**
*The guaranteed rate $1/2\lambda_{\max}\left(P\right)$ in Equation (22) is in general conservative. The true asymptotic decay rate of ∥x(t)∥ is −α(A), in the sense that $∥x\left(t\right)∥\leq C_{\varepsilon }\;e^{(\alpha (A)+\varepsilon )t}\;∥x\left(0\right)∥$ for every ε>0 and some $C_{\varepsilon }\geq 1$; for normal A one may take ε=0 and $C_{0}=1$. For the non-normal system matrices generated by asymmetric economic coupling, transient amplification with $C_{\varepsilon }>1$ is possible even when α(A)<0—an effect economically interpretable as short-run contagion overshoot preceding mean reversion, and indeed visible in the simulations of Section 7 (Manufacturing and Services overshoot their equilibria before relaxing; see Section 7.3).*

**Remark** **2** (Stochastic forcing)**.**
*In the presence of the noise term $\sigma_{ij}\xi_{ij}\left(t\right)$ in Equation (10), pointwise convergence to $x^{*}=0$ no longer holds. Instead, the system converges in distribution to a stationary Gaussian measure with covariance $\Sigma_{\infty }$ solving $A\Sigma_{\infty }+\Sigma_{\infty }A^{⊤}+D=0$, $D=\mathrm{diag}(\sigma_{ij}^{2})$, and the equilibrium is stable in the mean-square sense: $\lim_{t\to \infty }E{∥x\left(t\right)∥}^{2}=\mathrm{tr}\;\Sigma_{\infty }<\infty$. The deterministic results above apply to the noise-free skeleton $\sigma_{ij}=0$, which is the regime simulated in Section 7 and Section 8; Section 7.5 examines the behavior of the entropic diagnostics under non-trivial noise, where the stationary law is Gaussian rather than a point mass and both functionals acquire a strictly positive stationary floor.*

### 6.2. Economic Interpretation

Proposition 1 has a direct economic reading: the condition α(A)<0 is the requirement that channel friction rates $\Gamma_{ij}$ be collectively strong enough to overcome inter-sectoral propagation (the eigenvalues of $K_{\mathrm{flat}}$). When this condition fails—as in a systemic crisis where credit-channel coupling $K_{ijkl}$ becomes large relative to sectoral buffers $\Gamma_{ij}$—the equilibrium loses stability and the system undergoes a bifurcation toward persistent disequilibrium.

The Lyapunov function V provides a scalar measure of aggregate economic disequilibrium that is *dynamically consistent*: it decreases monotonically whenever the system is in its stable, unforced regime. This contrasts with ad hoc aggregate indicators (e.g., GDP gap) whose dynamics are not guaranteed to be Lyapunov functions of the underlying multi-dimensional system. Remark 1 adds a second layer to this reading: in non-normal economies, apparent short-run worsening (transient amplification of deviations in sectors far from the shock epicenter) is compatible with—indeed predicted by—global asymptotic stability.

## 7. Toy Model: A Stylized Financial-Contagion Scenario

### 7.1. Setup

We consider a 3×3 economy ($N_{S}=3$ sectors, $N_{A}=3$ agent classes) with sectors Manufacturing (M), Finance (F), and Services (S), and agent classes Households (H), Firms (Fi), and Government (G). The equilibrium capital-flow matrix (in normalized units) is

(24) $$M^{*}=\left(\begin{array}{ccc} 3.0 & 2.5 & 1.5\\ 1.5 & 4.0 & 1.0\\ 3.5 & 2.0 & 2.0 \end{array}\right),$$

where entry (i,j) represents the equilibrium flow from sector *i* to agent *j*. The channel-wise friction rates are

(25) $$\Gamma =\left(\begin{array}{ccc} 0.10 & 0.12 & 0.06\\ 0.15 & 0.18 & 0.08\\ 0.08 & 0.11 & 0.05 \end{array}\right),$$

corresponding to characteristic recovery times of $\tau_{ij}=\Gamma_{ij}^{-1}\approx 6$–20 quarters; recall from Equation (9) that these are assumed constant in time, and that the matrix entering *A* is diag(vec(Γ)).

The principal non-zero coupling entries of K are

(26) $$\begin{array}{cccc} K_{\left(M,\mathrm{Fi})(F,\mathrm{Fi}\right)} & =-0.08,\; & \; & \mathrm{credit}\;\mathrm{crunch} \end{array}$$

(27) $$\begin{array}{cccc} K_{\left(M,H)(F,\mathrm{Fi}\right)} & =-0.05,\; & \; & \mathrm{demand}\;\mathrm{shock} \end{array}$$

(28) $$\begin{array}{cccc} K_{\left(S,\mathrm{Fi})(F,\mathrm{Fi}\right)} & =-0.07,\; & \; & \mathrm{Finance}\;\mathrm{to}\;\mathrm{Services} \end{array}$$

(29) $$\begin{array}{cccc} K_{\left(S,\mathrm{Fi})(M,\mathrm{Fi}\right)} & =+0.03,\; & \; & \mathrm{supply-chain}\;\mathrm{spillover} \end{array}$$

(30) $$\begin{array}{cccc} K_{\left(M,G)(S,G\right)} & =+0.02,\; & \; & \mathrm{fiscal}\;\mathrm{stabilizer} \end{array}$$

where, following the target-first convention of Equation (8), the first index pair is the target channel and the second the source; the complete set of eleven entries is given in Table 3. The coupling is heuristically specified to represent the stylized transmission channels of 2007–2009; no empirical calibration is claimed (Section 3.2 and Section 10). The resulting system matrix *A* has spectral abscissa α(A)=−0.030<0, confirming stability, with asymptotic rate −α(A)=0.030 per quarter. The slowest mode of the system is the Services–Government channel, whose positive self-coupling (last row of Table 3) reduces its effective friction to 0.05−0.02=0.03 per quarter and thereby sets α(A): the long tail of the recovery is governed by the fiscal channel, not by the shocked credit channels. The Lyapunov matrix *P* obtained from Equation (19) has $\lambda_{\max}\left(P\right)=18.30$ and condition number κ(P)=7.6, so the guaranteed decay rate of V from Equation (21) is $1/\lambda_{\max}\left(P\right)=0.055$ per quarter, compared with the asymptotic rate 2|α(A)|=0.060 per quarter. The normalized commutator ${∥\left[A,A^{⊤}\right]∥}_{F}/{∥A∥}_{F}^{2}=0.19$ confirms that *A* is not normal, so the distinction drawn in Remark 1 is quantitatively relevant here.

The eleven entries represent the following stylized channels, each of which corresponds to a transmission mechanism documented in the literature surveyed in Section 2. The *credit crunch* (−0.08) is the contraction of bank lending to non-financial firms, the dominant channel in the 2007–2009 episode and the largest entry in the table; the *corporate lending* channel (−0.07) is its counterpart for services. *Consumer demand* (−0.05) and *household credit* (−0.04) transmit financial distress to manufacturing through the demand side, directly and through the contraction of household borrowing; *consumer credit* (−0.04) does the same for services. *Fiscal contraction* (−0.02) captures the reduction in government-intermediated flows that accompanies a financial contraction. The two positive real-sector entries are the *supply chain* (+0.03) and *demand spillover* (+0.02) couplings, by which manufacturing activity sustains service-sector flows—complementarity rather than contagion, consistent with the input–output evidence surveyed in Section 2.3 [2,24]. *Feedback to Finance* (−0.02) closes the loop from real-sector distress back to the financial sector through deteriorating loan quality. The two *fiscal stabilizer* entries (+0.02 each) are the only channels that act against the shock: government flows to services support manufacturing, and the self-coupling represents the automatic expansion of government transfers as activity falls. This last entry has a consequence worth flagging: by reducing the effective friction of the Services–Government channel it makes that channel the slowest mode of the system, so that in this calibration the long tail of the recovery is governed by the fiscal channel rather than by the shocked credit channels.

### 7.2. Shock Structure

The shock is modeled as a multi-component sustained forcing

(31) $$s_{ij}\left(t\right)=a_{ij}\;e^{-\delta_{ij}t},\;t\geq 0,$$

with amplitudes and decay rates representing a credit-market collapse that simultaneously impairs Finance–Firm credit ($a_{\mathrm{FFi}}=-0.50$, δ=0.12), Manufacturing–Firm investment ($a_{\mathrm{MFi}}=-0.20$, δ=0.15), Services–Firm lending ($a_{\mathrm{SFi}}=-0.18$, δ=0.15), and Manufacturing–Household demand ($a_{\mathrm{MH}}=-0.10$, δ=0.18). The Finance sector serves as the epicenter (largest amplitude, slowest decay), following the sequence observed in the 2007–2009 crisis.

### 7.3. Results: Propagation and Stability

**Figure 1:**
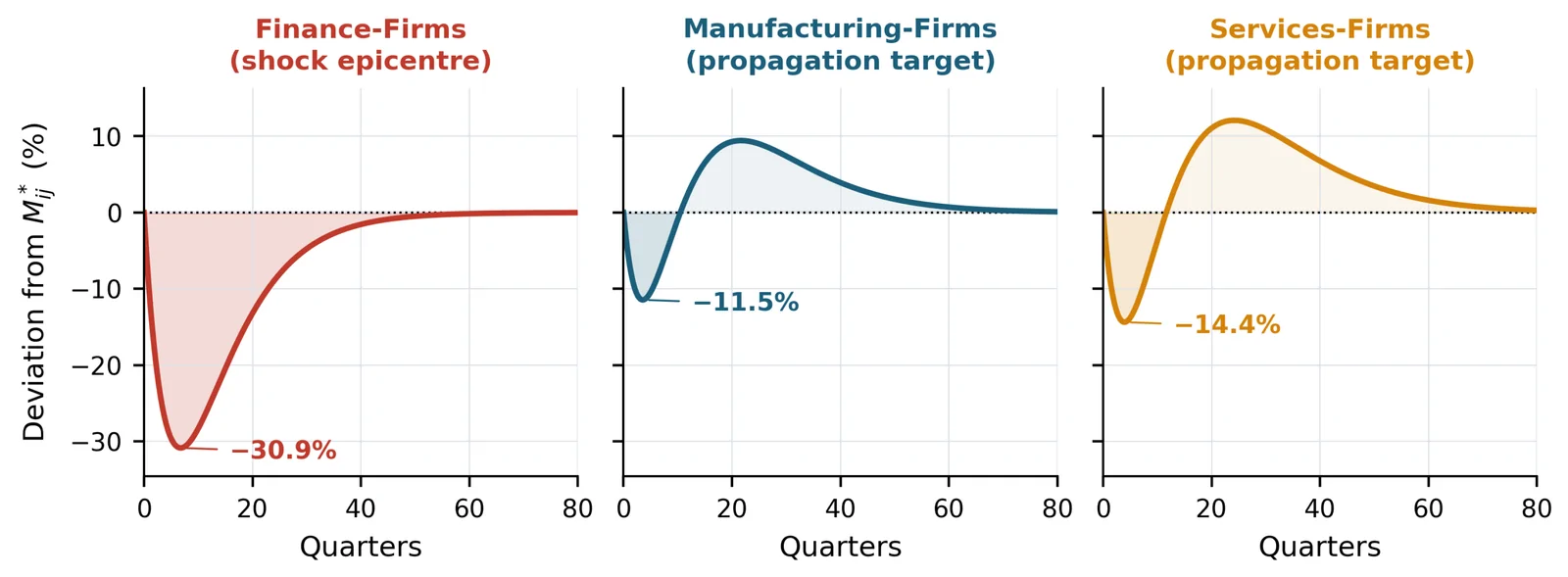
Monetary tensor $M_{ij}\left(t\right)$ under a stylized financial shock: the shock epicenter (Finance–Firms) and the two principal propagation targets (Manufacturing–Firms, Services–Firms), shown on a common vertical scale. The complete 3×3 array appears as Figure S1. Dotted horizontal lines denote equilibrium $M_{ij}^{*}$. Coloured shading highlights the deviation from equilibrium. Cross-sector propagation through K is visible in Manufacturing–Firms and Services–Firms. The overshoot above equilibrium in Manufacturing and Services is the transient amplification characteristic of non-normal *A* (Remark 1).

Figure 1 shows the three most affected components of the tensor $M_{ij}\left(t\right)$ under the shock; the complete 3×3 array is given as Figure S1 in the Supplementary Material. All nine components deviate from equilibrium, but with markedly heterogeneous amplitudes and recovery times. In this stylized calibration, Finance–Firms ($M_{22}$) experiences the deepest contraction (30.9% peak drop), followed by Services–Firms ($M_{32}$, 14.4%) and Manufacturing–Firms ($M_{12}$, 11.5%), in qualitative agreement with the cross-sector spillover pattern observed in 2007–2009 [2]. The overshoot of Manufacturing and Services above their equilibria before relaxation is the transient amplification predicted for non-normal *A* (Remark 1).

**Figure 2:**
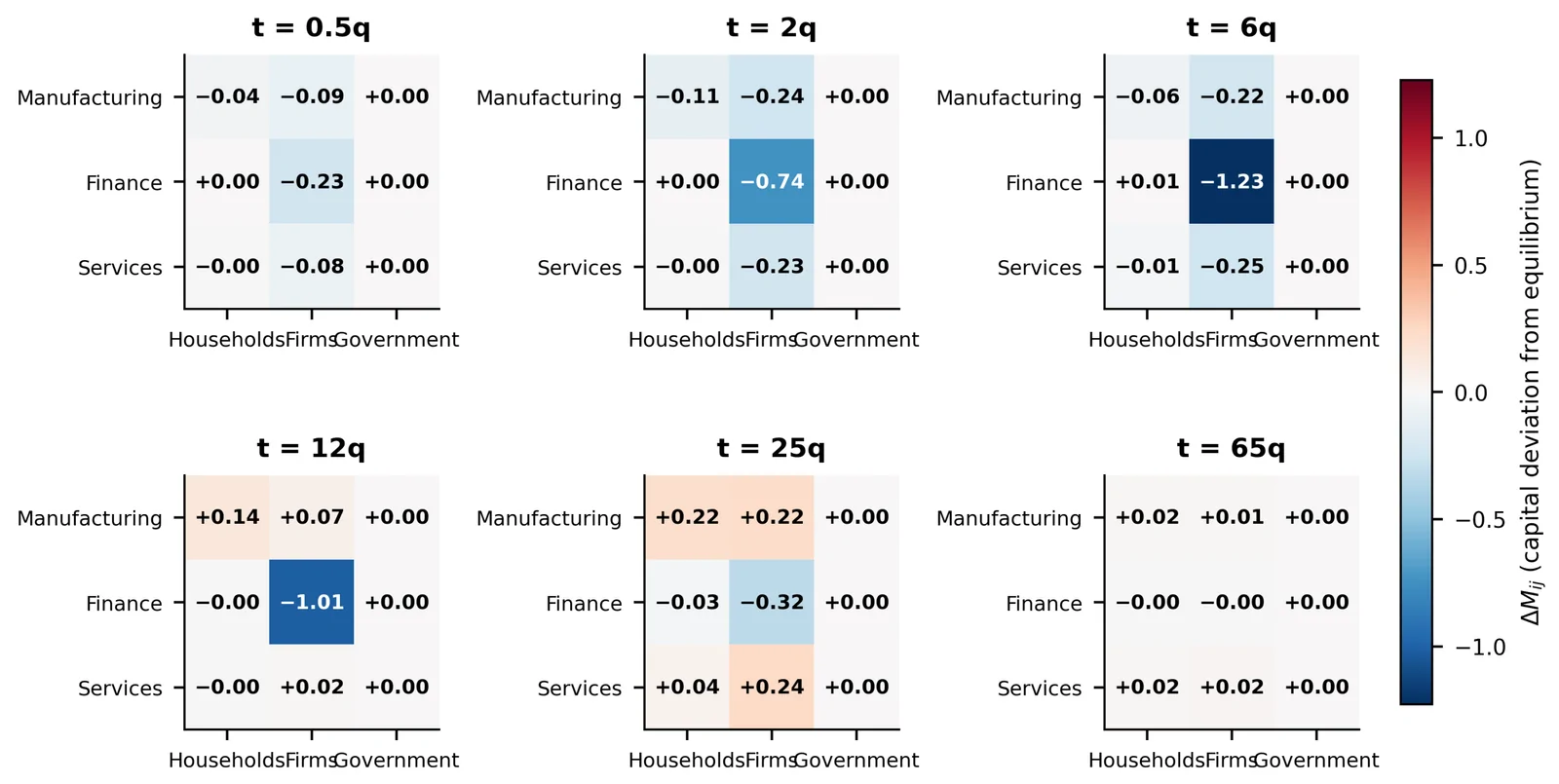
Deviation tensor $\Delta M_{ij}\left(t\right)$ at six temporal snapshots (t=0.5,2,6,12,25,65 quarters). Red: below equilibrium (capital deficit). Blue: above equilibrium (capital surplus). The shock originates at Finance–Firms (t=0.5 q) and progressively penetrates Manufacturing and Services through the coupling $K_{ijkl}$. By t=65 q the system has returned near equilibrium.

Figure 2 displays the deviation tensor $\Delta M_{ij}\left(t\right)=M_{ij}\left(t\right)-M_{ij}^{*}$ at six temporal snapshots. The propagation sequence is clear: at t=0.5 q, only the Finance sector is off-equilibrium; by t=6 q, Manufacturing and Services have accumulated significant deviations via the coupling K; by t=65 q, the system has nearly restored equilibrium.

**Figure 3:**
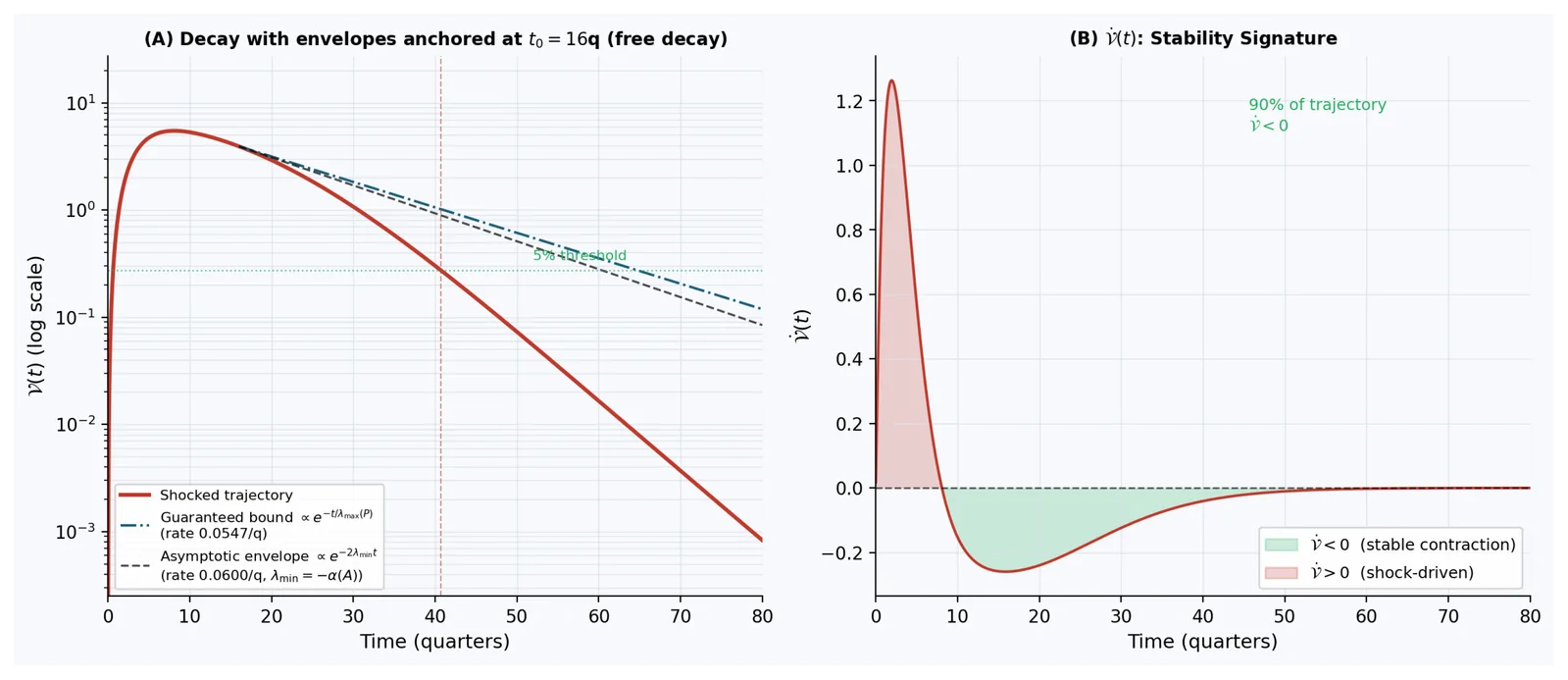
Lyapunov stability analysis. (**A**) $V\left(t\right)=x^{⊤}Px$ on a log scale. The dash-dotted curve is the guaranteed bound of Equation (21), rate $1/\lambda_{\max}\left(P\right)=0.055$/q; the dashed curve is the asymptotic envelope with rate 2|α(A)|=0.060/q. Both are anchored at $t_{0}=16$ quarters, in the free-decay regime where the residual forcing is negligible, and are strict majorants of the trajectory there. The dotted horizontal line marks the 5% threshold used to define the recovery time $\tau_{r}=40.7$ quarters (no policy). (**B**) $\dot{V}\left(t\right)$: positive only during the shock-driven phase (t<8 q), negative thereafter, confirming asymptotic stability.

Figure 3 confirms the Lyapunov analysis. Panel (A) shows V(t) on a log scale together with two envelopes anchored at $t_{0}=16$ quarters, where the residual shock forcing has become negligible: the guaranteed bound of Equation (21) with rate $1/\lambda_{\max}\left(P\right)=0.055$/q, and the asymptotic envelope with rate 2|α(A)|=0.060/q. Anchored in this free-decay regime, both curves are strict majorants of the empirical trajectory; anchored at the shock peak (t≈8 q) they would be mildly exceeded, because Proposition 1 applies to the unforced system while the exogenous forcing of Equation (31) is still active there. Panel (B) shows $\dot{V}\left(t\right)$: it is positive only during the driven phase (t<8 q), negative thereafter, confirming that the shock temporarily pumps energy into the system before mean-reversion takes over.

**Figure 4:**
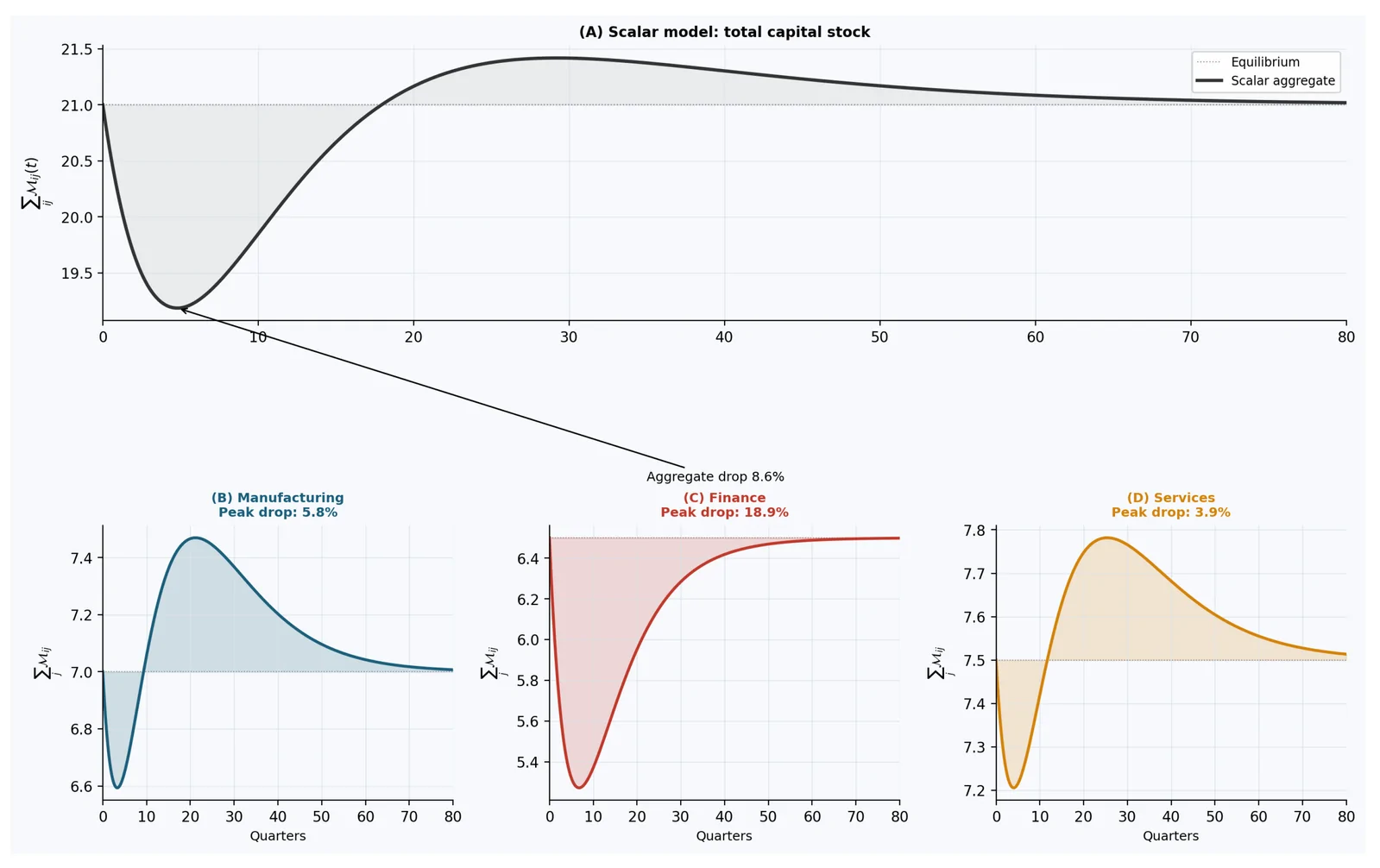
Scalar vs. tensor representation, in the stylized calibration of Section 7. (**A**) The scalar aggregate $\sum_{ij}M_{ij}\left(t\right)$ drops by 8.6% at its trough, masking the sectoral heterogeneity. (**B**–**D**) Per-sector capital totals $\sum_{j}M_{ij}\left(t\right)$ reveal peak drops of 5.8% (Manufacturing), 18.9% (Finance), and 3.9% (Services): the epicenter contracts by roughly twice the aggregate figure, while mildly affected sectors contract by less than half of it. Scalar aggregation therefore misrepresents where stabilization resources are needed during crises.

Figure 4 illustrates the information loss induced by scalar aggregation. In this calibration the aggregate capital stock $\sum_{ij}M_{ij}\left(t\right)$ drops by only 8.6% at its trough, while Finance suffers an 18.9% peak drop in total flows—a factor of roughly two of heterogeneity between the epicenter and the aggregate signal, with Manufacturing (5.8%) and Services (3.9%) in between. Scalar aggregation therefore substantially understates the damage at the epicenter while overstating it in mildly affected sectors; as Section 8.4 shows, this qualitative pattern holds in every draw of the perturbed ensemble, and it is the fundamental motivation for the tensor representation.

### 7.4. Results: Entropic Diagnostics

**Figure 5:**
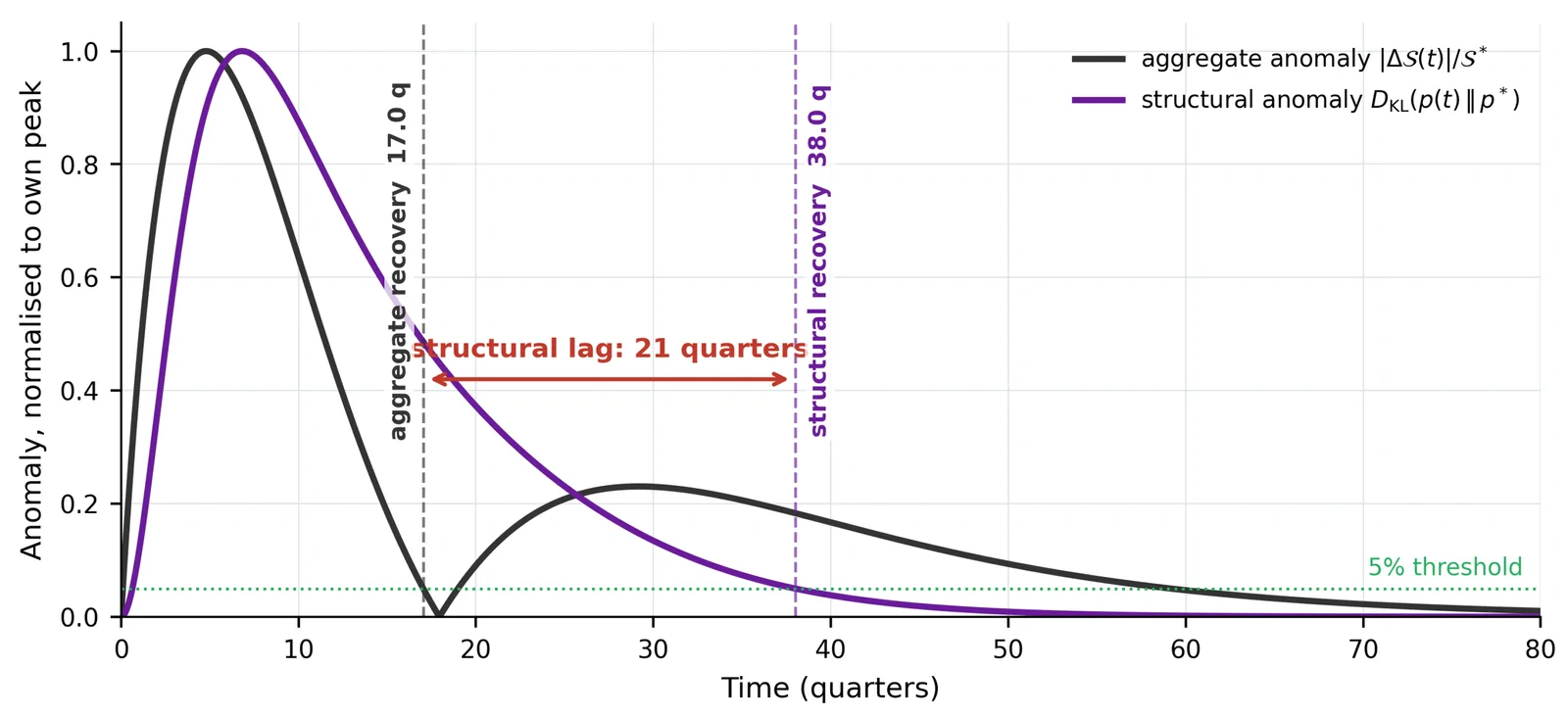
Aggregate recovery precedes structural recovery. Aggregate anomaly $\left|\Delta S\right|/S^{*}$ and structural anomaly $D_{\mathrm{KL}}$, each normalized to its own peak: in this stylized calibration, the aggregate recovers below the 5% threshold in 17.0 quarters, the structure only in 38.0 quarters, a lag of 21 quarters. This is a property of the present calibration only: across the Monte Carlo ensemble of Section 8.4, the sign of the lag is positive in 96.6% of draws, but its magnitude ranges from 1.8 to 34.7 quarters between the 5th and 95th percentiles (Section 8.4), so the 21-quarter value shown here should not be read as representative.

Figure 5 reports the information-theoretic observables of Section 5 along the shocked trajectory. All flows remain strictly positive throughout ($\min_{ij,t}M_{ij}\left(t\right)=1.0$), so Equations (14)–(18) are well defined.

**Figure 6:**
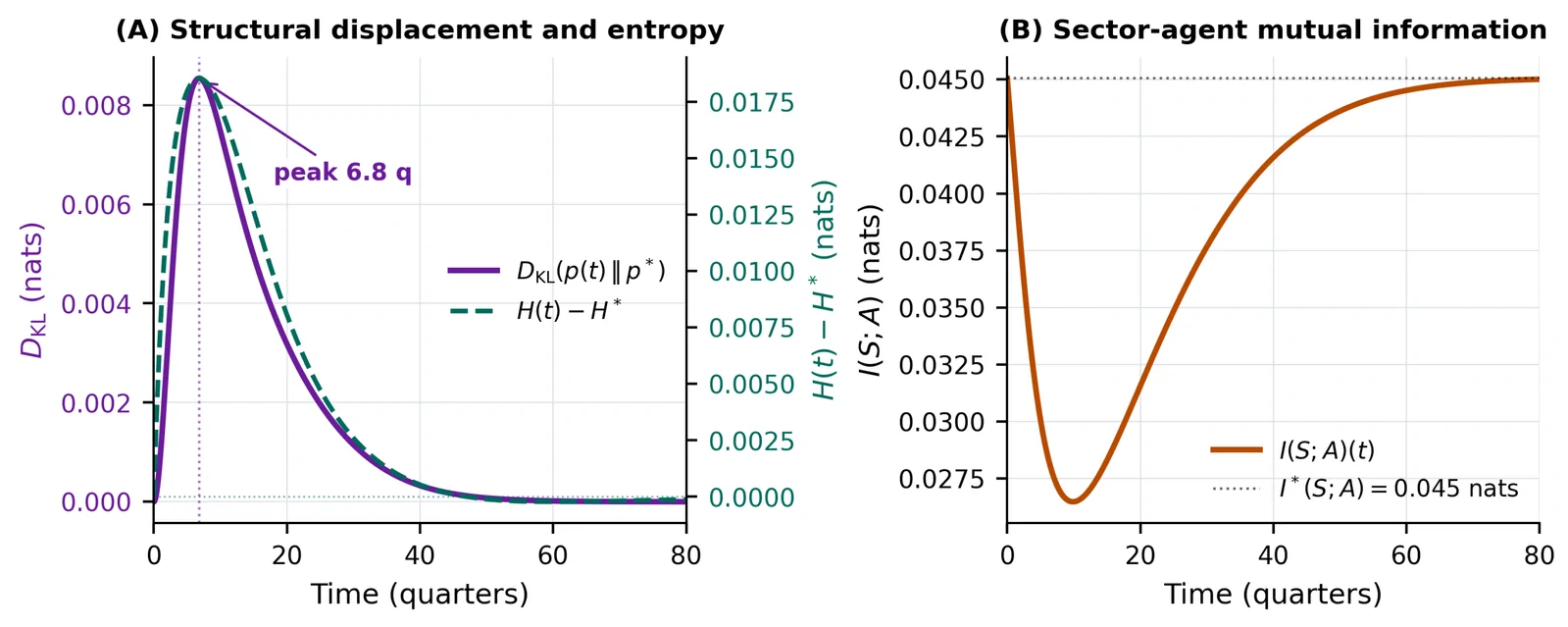
Auxiliary entropic diagnostics. (**A**) Structural displacement $D_{\mathrm{KL}}\left(p\left(t\right)\;∥\;p^{*}\right)$ (left axis), peaking at t=6.8 q, and entropy deviation $H\left(t\right)-H^{*}$ (right axis), with $H^{*}=2.116$ nats. (**B**) Sector–agent mutual information I(S;A)(t); its strictly positive equilibrium value $I^{*}\left(S;A\right)=0.045$ nats certifies that the rank-1 (separable) approximation is information-theoretically insufficient.

The structural displacement $D_{\mathrm{KL}}\left(p\left(t\right)\;∥\;p^{*}\right)$ peaks at t=6.8 quarters (value $8.5\times 10^{-3}$ nats, corresponding to a reallocation share $\delta_{\max}=5.2\%$), while the entropy deviation $H\left(t\right)-H^{*}$ is measured about the equilibrium value $H^{*}=2.116$ nats (the maximum possible for nine channels being ln9=2.197 nats) (Figure 6). The central result of this section is the comparison of recovery times: normalizing the aggregate anomaly $\left|\Delta S\left(t\right)\right|/S^{*}$ and the structural anomaly $D_{\mathrm{KL}}\left(t\right)$ each to their own peaks, in this calibration the aggregate recovers below the 5% threshold in 17.0 quarters, whereas the structure requires 38.0 quarters—*a lag during which a scalar observer would declare the crisis essentially over while the sectoral composition of capital flows remains far from equilibrium*. We report the magnitude of this lag (21 quarters) as a property of the present calibration only: Section 8.4 shows that while the lag is positive in 96.6% of the perturbed ensemble, its magnitude ranges from 1.8 to 34.7 quarters between the 5th and 95th percentiles, so the sign rather than the magnitude is the robust finding. Finally, the sector–agent mutual information has equilibrium value $I^{*}\left(S;A\right)=0.045$ nats, strictly positive, certifying that $M^{*}$ is not rank-1 and that the separable approximation (7) is information-theoretically insufficient; the crisis perturbs I(S;A) by up to 0.019 nats before it relaxes back to $I^{*}$.

### 7.5. Diagnostics Under Stochastic Forcing

The diagnostics above are computed along deterministic trajectories, whereas Equation (10) admits a stochastic forcing term. This matters because both $D_{\mathrm{KL}}$ and I(S;A) are nonlinear in *p*: under σ≠0 the stationary law is Gaussian with covariance $\Sigma_{\infty }$ (Remark 2) rather than a point mass at equilibrium, so the diagnostics acquire a strictly positive stationary floor against which any crisis signal must be read.

We therefore repeat the analysis under noise. For $\sigma_{ij}=\sigma \;M_{ij}^{*}$ with σ∈{0.005,0.01,0.02} we integrate the Itô system by Euler–Maruyama (Δt=0.01 q) over 400 paths per noise level, compute the path-wise diagnostics, and report ensemble means with 5th–95th bands together with the stationary floor implied by $\Sigma_{\infty }$. Table 4 collects the results.

The crisis signal survives at all three noise levels, but with a rapidly shrinking margin. The peak ensemble-mean divergence is essentially unchanged from its deterministic value ($8.5\times 10^{-3}$ nats), whereas the stationary floor grows as $\sigma^{2}$, so the peak-to-floor ratio falls from 109 at σ=0.005 to 7.9 at σ=0.02. The structural lag remains positive in 100%, 100% and 99.2% of paths respectively, with the median lag rising from 21.1 to 30.1 quarters as noise inflates the time taken for the divergence to fall below a threshold defined relative to its own peak. The qualitative conclusion of Section 7.4 therefore holds under stochastic forcing over the range examined; extrapolating beyond σ=0.02 would not be warranted, since at that level the floor is already within an order of magnitude of the signal.

## 8. Tensor-Guided Policy Design

### 8.1. Policy as a Forcing Term

A fiscal or monetary policy intervention enters the dynamics as a time-varying forcing $s^{\mathrm{pol}}\left(t\right)$ added to the right-hand side of Equation (13):

(32) $$\dot{x}=Ax+s^{\mathrm{shock}}\left(t\right)+s^{\mathrm{pol}}\left(t\right).$$

The matrix $B\in R^{(N_{S}N_{A})\times m}$ appearing below is the *policy transmission matrix*: its columns span the channels through which the authority can act, and its entry $B_{aq}$ gives the effect on the deviation of channel *a* of a unit of instrument *q*, with *m* the number of independent instruments. The economic content of *B* is the set of interventions available: an authority able to direct expenditure to any sector–agent cell has $m=N_{S}N_{A}$ and B=I, whereas one restricted to, say, corporate credit support and household transfers has m=2 and a *B* whose columns are supported on the corresponding cells. Throughout the benchmark of Section 8.5 we set B=I. This is the most permissive specification and therefore defines a genuine upper bound on attainable performance: no more restricted instrument set can do better, so the frontier we compute is a conservative standard against which to judge the heuristics.

The optimal policy minimizes a cost functional $C=\int_{0}^{\infty }\left[V\left(x\left(t\right)\right)+{\rho ∥s^{\mathrm{pol}}\left(t\right)∥}^{2}\right]\;dt$, which balances integrated disequilibrium (as measured by V) against policy expenditure, with ρ>0 a budget parameter. This is a standard linear-quadratic regulator (LQR) problem with state cost Q=P and control cost R=ρI [42] (Ch. 3). Since α(A)<0, the pair (A,B) is stabilizable and, as P≻0, the pair $\left(A,Q^{1/2}\right)$ is detectable, so the algebraic Riccati equation

(33) $$A^{⊤}S+SA-\rho^{-1}SBB^{⊤}S+P=0$$

admits a unique symmetric positive-semidefinite stabilizing solution [43] (Ch. 7), and the optimal policy is the linear feedback

(34) $$s^{\mathrm{pol}}\left(t\right)=-\rho^{-1}B^{⊤}S\;x\left(t\right).$$

Equation (33) is solved numerically by the Schur method of Laub [39]. We emphasize that the LQR rule serves as a theoretical benchmark: it presupposes complete knowledge of the system matrix *A* (hence of *P* and *S*), continuous observation of the full tensor state, and the ability to both inject and withdraw capital in every sector–agent channel. The two rules compared in Section 8.2 are deliberately simpler, implementable heuristics; Section 8.5 quantifies how close each comes to the LQR benchmark.

### 8.2. Uniform vs. Targeted Stimulus Under a Symmetric Exit Rule

In practice, policymakers rarely implement the optimal LQR rule. We compare two stylized alternatives, defined formally in Section 3.4:

- **Uniform (scalar-information) stimulus:** injection proportional to equilibrium shares, $s_{ij}^{\mathrm{uni}}\propto M_{ij}^{*}$, Equation (1);
- **Targeted (tensor-information) stimulus:** injection proportional to the current *capital deficit* of each sector–agent pair, $s_{ij}^{\mathrm{tgt}}\propto \max\left(-x_{ij},\;0\right)$, Equation (3).

Two specifications of these rules must be distinguished, and we make the terminating one primary because it is the controlled comparison. Under the *symmetric exit rule*, both policies are active only on the window $W=[t_{0},\;t_{0}+T_{\mathrm{pol}}]$ with $T_{\mathrm{pol}}=8$ quarters, so that termination is imposed identically on both and any difference in outcome is attributable to the allocation rule alone. Under the *non-terminating specification*, the uniform rule decays slowly but never switches off, whereas the targeted rule self-terminates as deficits close; that comparison therefore conflates targeting with termination, and we report it separately in Section 8.3 as an illustration of the informational asymmetry between a scalar and a tensor policy authority rather than as a horse race between two rules.

A remark on comparability is required, and it turns out to strengthen rather than qualify the result. At the common rate λ=0.25 the uniform rule spends its full nominal budget $\lambda T_{\mathrm{pol}}=2.0$, whereas the targeted rule spends only 1.65, because it self-terminates once all deficits close inside the window (at t=14.60 quarters, i.e., after 6.60 of the 8 available quarters). (This figure corrects a value of 1.75 reported in the previously submitted version. That value was a numerical artifact: the injection rate was evaluated on a fixed grid, and the rule was treated as active whenever the total deficit exceeded $10^{-10}$, a threshold below the floating-point residue that accumulates once the deficits have genuinely closed. Nineteen grid samples after true closure were consequently counted as active, contributing a spurious 19×Δt×λ=0.095. Locating the closure by event detection instead of by threshold on a grid gives λ×(14.60−8)=1.65, which the fixed-grid computation also returns once the threshold is raised above the noise floor. Only this expenditure integral is affected; all recovery times and trajectories are unchanged). The two are therefore not budget-neutral, and one might expect the imbalance to be removable by raising $\lambda^{\mathrm{tgt}}$. It is not. Realized expenditure of the targeted rule is non-monotone in the rate and saturates: sweeping $\lambda^{\mathrm{tgt}}\in \left[0.20,\;1.20\right]$, gross expenditure peaks at 1.70 near $\lambda^{\mathrm{tgt}}=0.40$ and falls thereafter, because a larger injection closes the deficits sooner and shortens the active period. The targeted rule *cannot* spend the uniform rule’s budget within this window.

The correct statement of the comparison is therefore not that the two policies are budget-matched but that the targeted rule dominates while spending strictly less: at its maximum attainable expenditure ($\lambda^{\mathrm{tgt}}=0.40$, gross 1.70, i.e., 85% of the uniform budget) it recovers in 11.1 quarters against the uniform rule’s 45.1, a reduction of 75%. The baseline comparison at equal *rate* (12.8 against 45.1 quarters) is thus conservative in the targeted rule’s favor on the expenditure dimension. Gross and net expenditure are reported for every policy so that this can be verified directly rather than reconstructed from the text.

**Figure 7:**
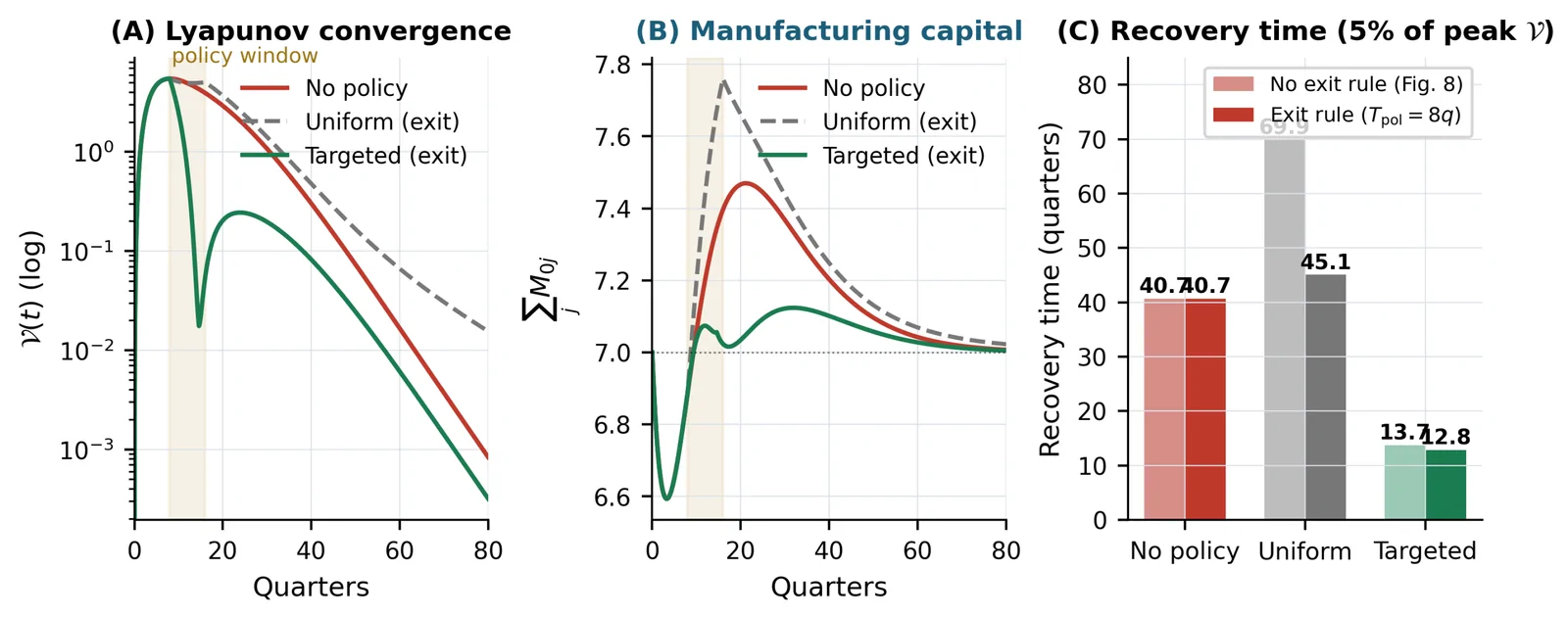
Primary policy comparison, under a symmetric exit rule: both policies are active only for $t_{0}\leq t\leq t_{0}+T_{\mathrm{pol}}$ with $T_{\mathrm{pol}}=8$ quarters and the same nominal rate λ=0.25, hence the same nominal budget $\lambda T_{\mathrm{pol}}=2.0$; realized gross expenditure differs (2.0 uniform against 1.65 targeted), as discussed in Section 8.2. (**A**) Lyapunov convergence; the shaded band marks the policy window. (**B**) Manufacturing capital stock. (**C**) Recovery times, shown as paired bars for each scenario: within each pair the lighter shade is the non-terminating specification of Section 8.3 and the darker shade the symmetric exit rule used here ($T_{\mathrm{pol}}=8$ q); red denotes no policy, gray the uniform rule and green the targeted rule. In this stylized calibration, the uniform policy recovers in 45.1 quarters (+11% vs. no policy) and the targeted policy in 12.8 quarters—72% faster than uniform under identical termination.

Figure 7 presents the results at the common rate λ=0.25:

- **No policy:** recovery in $\tau_{r}=40.7$ quarters;
- **Uniform (exit rule):** recovery in $\tau_{r}=45.1$ quarters (+11% vs. no policy);
- **Targeted (exit rule):** recovery in $\tau_{r}=12.8$ quarters (−69% vs. no policy).

In this calibration, the targeted policy is thus 72% faster than the uniform policy when both are subject to the same termination rule: *the advantage is attributable to targeting, not to termination*. Section 8.4 shows that the ordering holds in 100% of the perturbed ensemble.

The mechanism is a direct consequence of the tensor structure. A share-weighted injection adds capital to channels that are near or above equilibrium (Government channels, near-equilibrium Services), creating positive deviations that increase V and slow convergence. The targeted policy, by construction, directs resources exclusively toward components with $x_{ij}<0$, driving all deviations toward zero simultaneously. This parallels a familiar result in nonlinear control: a globally stabilizing control must respect the geometry of the Lyapunov level sets, which in general are not spherical. Uniform forcing ignores that geometry; targeted forcing aligns with it.

### 8.3. Secondary Experiment: Scalar Versus Tensor Information

The comparison above imposes a common exit rule on both authorities. It is also instructive to consider the case in which the exit rule itself is informationally constrained. An authority that observes only the aggregate S(t) has no signal on which to condition sector-level termination, whereas the targeted policy self-terminates as deficits close, since $s_{ij}^{\mathrm{tgt}}\to 0$ as $x_{ij}\to 0^{-}$. Under the non-terminating specification of Equation (2), the uniform rule therefore continues to inject capital into components that have already returned to—or overshot—their equilibrium values.

**Figure 8:**
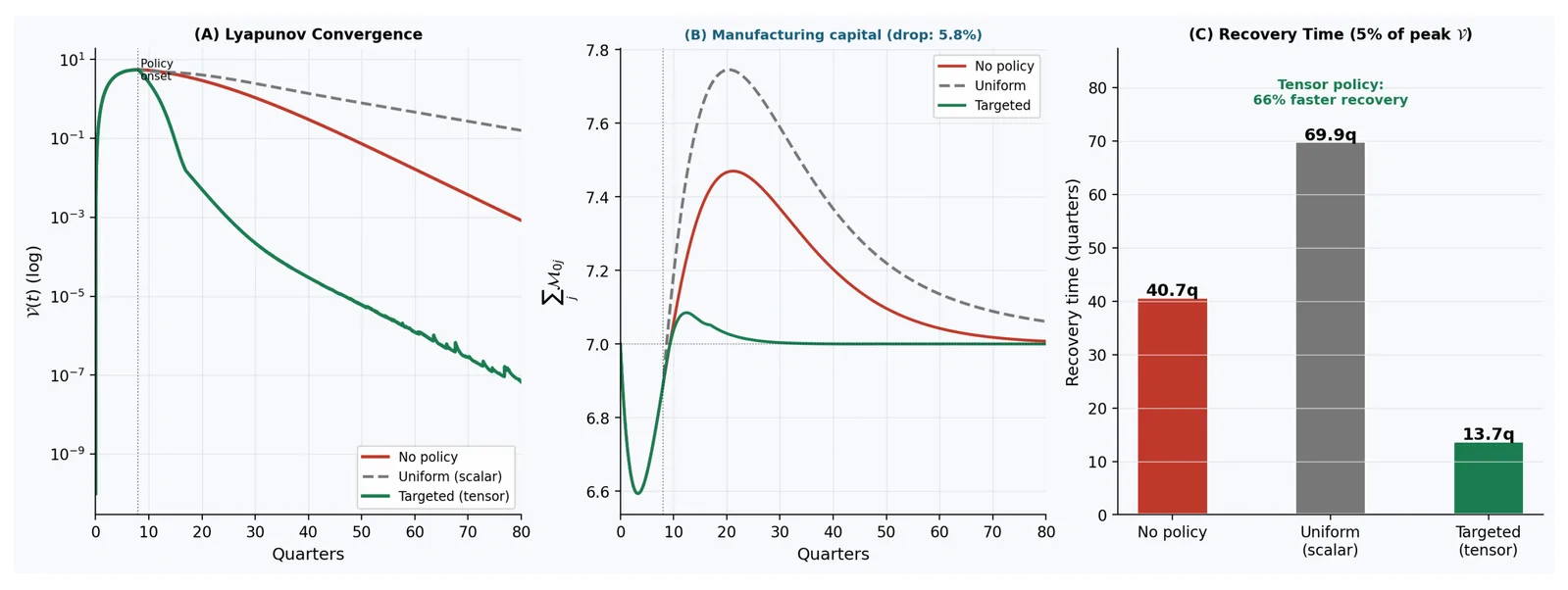
Secondary experiment: non-terminating uniform stimulus versus self-terminating targeted stimulus, equal rate λ=0.25 deployed at $t_{0}=8$ quarters post-shock. (**A**) Lyapunov function V(t) under no policy (red), uniform stimulus (grey dashed), and targeted tensor stimulus (green). (**B**) Manufacturing capital stock $\sum_{j}M_{0j}\left(t\right)$. (**C**) Recovery times $\tau_{r}$. The extreme delay of the uniform rule here reflects the informational asymmetry between the two authorities—an authority observing only the aggregate has no signal on which to condition termination, whereas the targeted rule self-terminates—rather than any general tendency of share-weighted allocation to be counterproductive; see Section 8.2 for the controlled comparison under a common exit rule.

Figure 8 presents this comparison:

- **No policy:** recovery in $\tau_{r}=40.7$ quarters;
- **Uniform stimulus (non-terminating):** recovery in $\tau_{r}=69.9$ quarters—*slower* than no policy (+72%);
- **Targeted stimulus:** recovery in $\tau_{r}=13.7$ quarters—66% faster than no policy.

Comparison with Section 8.2 isolates the two effects: of the uniform policy’s delay, the part that survives the imposition of a symmetric exit rule (+11%) is attributable to allocation, and the remainder (+72% against +11%) to the absence of termination. We stress that this experiment should be read as *scalar-information policy versus tensor-information policy*, and not as evidence that share-weighted allocation is intrinsically counterproductive.

### 8.4. Robustness: Monte Carlo Sensitivity Analysis

The quantitative results above are computed on a single, heuristically specified calibration. To assess whether the paper’s conclusions are artifacts of that particular parameter choice, we repeat the entire analysis on the ensemble of N=1000 perturbed economies specified in Section 3.7. All 1000 draws satisfy the stability condition α(A)<0 (spectral abscissae in [−0.055,−0.006]) and all maintain strictly positive flows, so that Equations (14)–(18) remain well defined.

**Figure 9:**
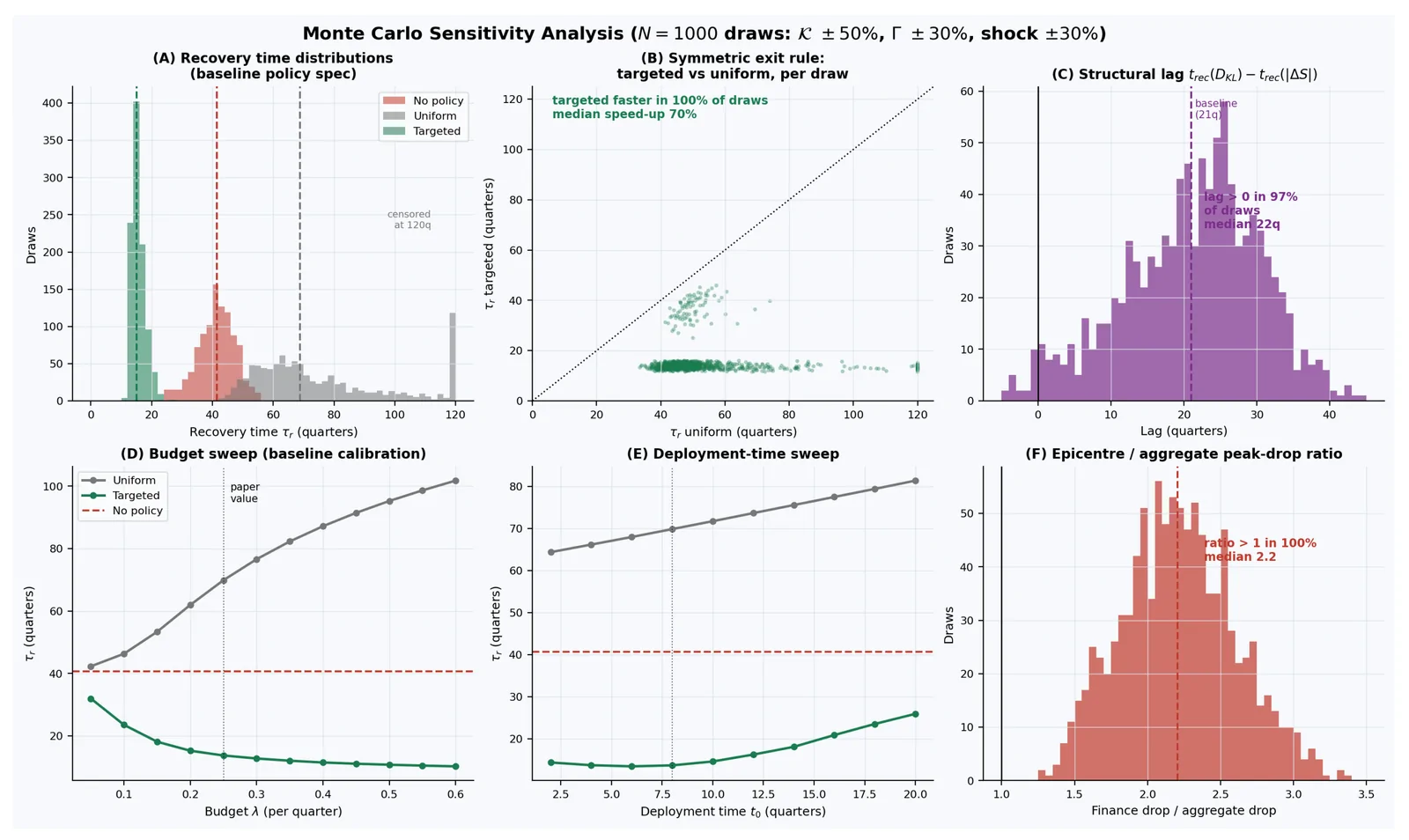
Monte Carlo sensitivity analysis (N=1000 draws; K ±50%, Γ ±30%, shocks ±30%). (**A**) Recovery-time distributions under the non-terminating policy specification; dashed lines mark medians; the uniform-policy mass at 120 quarters is right-censored. (**B**) Per-draw recovery times under the symmetric exit rule: every draw lies below the diagonal, i.e., targeting beats uniformity in 100% of the ensemble (median speed-up 70%). (**C**) Distribution of the structural lag; positive in 96.6% of draws (median 22 quarters; full range in Table 5). (**D**,**E**) One-at-a-time sweeps of the budget λ and the deployment time $t_{0}$ on the baseline calibration. (**F**) Epicenter-to-aggregate peak-drop ratio; greater than one in 100% of draws (median 2.2).

Table 5 and Figure 9 summarize the outcome. The point estimates reported above are, as expected, not robust as numbers: recovery times, peak drops, and the structural lag all vary substantially across the ensemble (e.g., the no-policy recovery time spans 30–50 quarters between the 5th and 95th percentiles). The paper’s three *qualitative* conclusions, however, are preserved in essentially the entire ensemble:

1. *Aggregation hides the epicenter.* The Finance peak drop exceeds the scalar-aggregate drop in 100% of draws, with a median epicenter-to-aggregate ratio of 2.2 (5th–95th percentiles: 1.6–2.9); the factor-of-two heterogeneity of Figure 4 is thus a generic property of the coupled system, not of the particular calibration.
2. *Structure recovers after the aggregate.* The structural lag $t_{\mathrm{rec}}\left(D_{\mathrm{KL}}\right)-t_{\mathrm{rec}}\left(|\Delta S|\right)$ is positive in 96.6% of draws (median 22 quarters) and exceeds two years in 88.9% of draws. Its dispersion, however, is very wide: the 5th–95th percentile range is 1.8–34.7 quarters, spanning nearly two orders of magnitude. The  21-quarter figure of Section 7.4 is therefore one draw from a broad distribution and is not representative of the model’s behavior across calibrations. What is robust is the sign of the lag, not its size, and we state the conclusion in those terms throughout.
3. *Targeting beats uniformity.* Under the symmetric exit rule, which controls for termination effects, the targeted policy is faster than the uniform policy in 100% of draws, with a median speed-up of 70% (Figure 9B). Under  the non-terminating specification, the targeted policy recovers faster than no policy in 100% of draws and the full ordering $\tau_{r}^{\mathrm{tgt}}<\tau_{r}^{\mathrm{none}}<\tau_{r}^{\mathrm{uni}}$ holds in 99.4%.

One-at-a-time sweeps on the baseline calibration (Figure 9D,E) show in addition that the policy ordering is preserved for every budget λ∈[0.05,0.6], every deployment time $t_{0}\in \left[2,\;20\right]$ quarters, and every recovery threshold between 2% and 10% of the peak: under the non-terminating specification, the targeted recovery time decreases monotonically with the budget while the uniform recovery time *increases* monotonically with it, consistent with the geometric interpretation above. The only notable caveat is one of magnitude, not sign: in 11.6% of draws the non-terminating uniform policy has not recovered by the 120-quarter horizon (right-censored in Table 5), so its reported dispersion is a lower bound.

We conclude that the headline numbers of the paper should be read as illustrative values of a stylized calibration, as stated in Section 3.2 and Section 10, but that the ordinal conclusions—aggregation masks the epicenter, structure lags the aggregate, and deficit-targeted stimulus dominates uniform stimulus—are robust across a wide neighborhood of the calibration.

### 8.5. Optimal-Control Benchmark

To position the two heuristics against the optimum, we solve the LQR problem of Section 8.1 exactly and simulate the Riccati feedback $u\left(t\right)=-\rho^{-1}B^{⊤}S\;x\left(t\right)$, which for the choice B=I made here reduces to $u\left(t\right)=-\rho^{-1}S\;x\left(t\right)$, activated at the same deployment time $t_{0}=8$ quarters as the heuristics, for a grid of budget parameters ρ. Because the feedback rule has no fixed budget, comparability requires a common expenditure measure; we use the gross expenditure $\int \sum_{ij}\left|u_{ij}\left(t\right)\right|\;dt$ (for the one-sided heuristics this coincides with total injected capital) and, on the cost side, the decomposition of C into integrated disequilibrium $J_{V}=\int V\;dt$ and control effort $J_{u}=\int {∥u∥}^{2}\;dt$.

**Figure 10:**
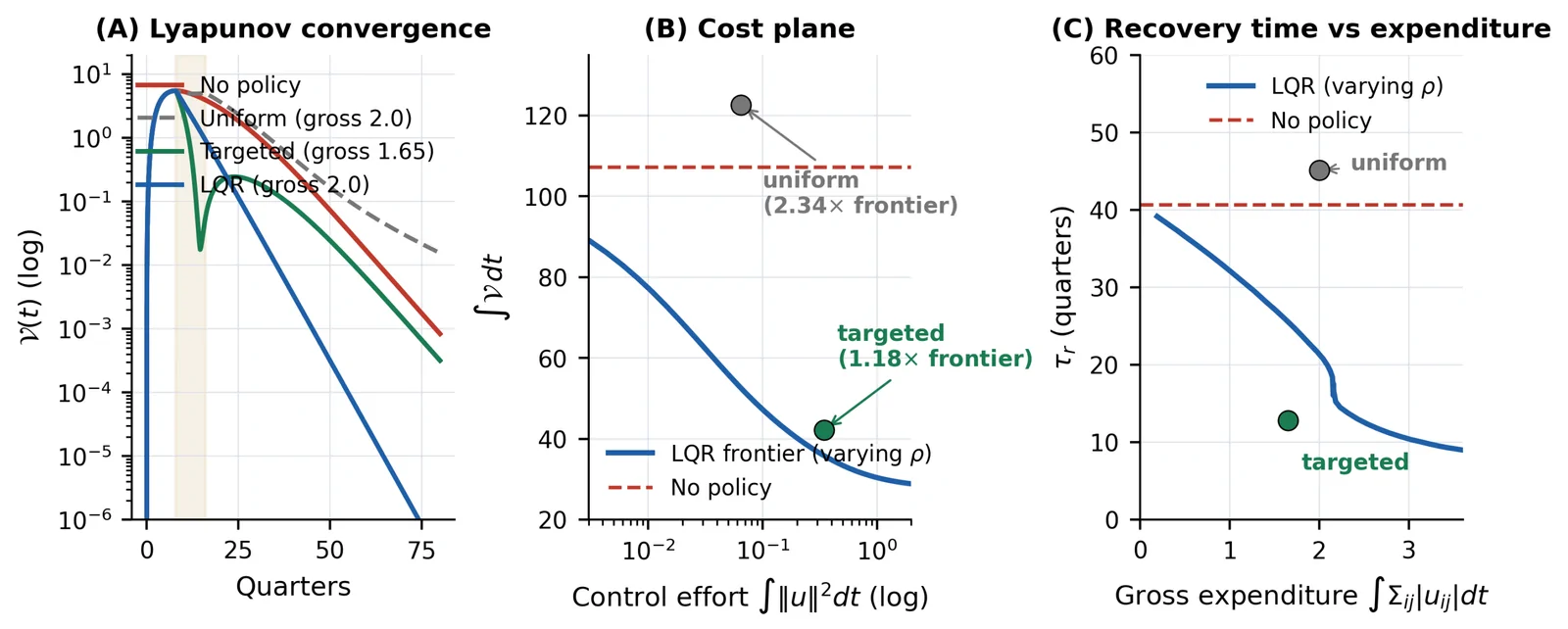
Optimal-control (LQR) benchmark, with the Riccati feedback $u=-\rho^{-1}B^{⊤}S\;x$ deployed at $t_{0}=8$ quarters and B=I. (**A**) Lyapunov convergence for no policy, the two exit-rule heuristics, and the budget-matched LQR ($\rho^{*}=128$, gross expenditure 2.0); the shaded band marks the heuristic policy window. (**B**) Cost plane: integrated disequilibrium $J_{V}=\int V\;dt$ versus control effort $J_{u}=\int {∥u∥}^{2}\;dt$. The LQR traces the Pareto frontier; at equal effort the targeted heuristic is 1.18× the frontier value, while the uniform heuristic is 2.34×. (**C**) Recovery time versus gross expenditure. The LQR reaches the threshold later than the targeted heuristic because it minimizes integrated cost with two-sided flows rather than time-to-threshold; this is a difference in objective, not performance.

Figure 10 reports two complementary comparisons, both under the symmetric exit rule of Section 8.2 for the heuristics.

*Cost plane (Figure 10B).* As ρ varies, the LQR traces the Pareto frontier in the $\left(J_{u},J_{V}\right)$ plane: no policy of equal control effort can achieve lower integrated disequilibrium. At equal effort, the targeted heuristic attains $J_{V}=42.2$ against a frontier value of 35.7—only 18% above the optimum—despite requiring no knowledge of *A*, *P*, or *S*, but only observation of the current deficits. The uniform heuristic attains $J_{V}=122.7$ against a frontier value of 52.5 (2.34× the optimum) and is in this calibration dominated by doing nothing ($J_{V}^{\mathrm{none}}=107.3$). This is the like-for-like comparison, because $J_{V}$ at given $J_{u}$ is the quantity the LQR actually optimizes.

*Operational metric (Figure 10A,C).* At matched gross expenditure (2.0), the budget-matched LQR ($\rho^{*}=128$) reaches the recovery threshold in 21.3 quarters, later than the targeted heuristic (12.8). This is a mismatch of metrics and not a performance ranking, and we want to be explicit that it should not be read as one: the LQR minimizes the integrated cost C, not the time at which V first crosses a threshold, and it is optimal for the former by construction. The threshold-crossing metric $\tau_{r}$ rewards front-loaded, all-positive injections, whereas the LQR intervenes on both sides—its net expenditure is only 0.04 against gross expenditure of 2.0, so it withdraws from overshooting channels almost as much as it injects into deficient ones. A time-optimal formulation, or an LQR with a terminal penalty, would be expected to dominate the heuristic on $\tau_{r}$; we have not constructed one, and we therefore make no claim that the heuristic outperforms optimal control on any metric.

Together, the three policies form an information ladder: the uniform rule uses only scalar information; the targeted rule uses the tensor state and captures most of the optimal-control gain; the LQR additionally uses the full model (A,P,S) and defines the attainable frontier. The proximity of the targeted heuristic to that frontier is the operative policy message: *most of the value of optimal control is already obtained by observing the disaggregated state*, which is precisely the information destroyed by scalar aggregation (Section 5).

### 8.6. Structural Implications for Policy Design

The toy model suggests three principles for tensor-informed policy:

1. **Target deficits, not flows.** Effective stimulus should be proportional to $\max(-x_{ij},\;0)$, not to $M_{ij}^{*}$. This requires real-time sectoral accounting at the sector–agent resolution of the tensor—a capability increasingly feasible with modern administrative data [30,44], subject to the governance constraints of Section 10.2.
2. **Respect the coupling structure.** Because $K_{\left(M,\mathrm{Fi})(F,\mathrm{Fi}\right)}<0$, restoring Finance–Firms does not automatically restore Manufacturing–Firms: the credit channel must also be directly addressed. Policy conditioned only on aggregate credit will, within this model, systematically under-restore real-sector flows.
3. **Monitor V(t) and $D_{\mathrm{KL}}\left(t\right)$, not only GDP.** The Lyapunov function integrates the full tensor deviation into a single real-time indicator of systemic disequilibrium that is dynamically consistent with the model’s recovery geometry, and the KL divergence of Section 5 isolates the *structural* component of the anomaly. GDP-like aggregates, by contrast, may signal recovery substantially before the sectoral structure has actually normalized (Figure 5).

## 9. Discussion

We situate each of the paper’s three substantive claims against the literatures of Section 2, and state in each case what we take the marginal contribution to be.

*Aggregation masks the epicenter.* That aggregate fluctuations have disaggregated origins is established by the granularity and production-network literatures [2,23,24], and the finding of Section 7—an epicenter contraction roughly twice the aggregate—is consistent with them rather than novel against them. The marginal contribution is the measurement: the masking is expressed in information-theoretic units and shown, in Section 8.4, to hold in 100% of a perturbed ensemble, which establishes it as a property of the coupled structure rather than of the particular parameterization.

*Structure recovers after the aggregate.* This connects to the distinction between contagion and interdependence drawn by Forbes and Rigobon [19] and, more loosely, to the literature on compositional recoveries. The marginal contribution is a state-dependent diagnostic, $D_{\mathrm{KL}}\left(p\left(t\right)∥p^{*}\right)$, whose recovery time is directly comparable with that of the aggregate, so that the disagreement between the two observables becomes measurable. We are careful about the strength of this result: as Section 8.4 shows, the *sign* of the lag is robust (96.6% of draws) while its *magnitude* is not (5th–95th percentiles 1.8–34.7 quarters). The defensible claim is therefore that structure recovers later than the aggregate, not that it recovers twenty-one quarters later.

*Targeting dominates uniformity.* The result is an instance of the general problem of policy under partial observation [33,34,35], and Bernanke’s [36] assessment of the declining informativeness of monetary aggregates is the closest policy statement of the same point. The marginal contribution is the decomposition against an exact optimal-control frontier: the targeted heuristic comes within 18% of the frontier at equal effort while requiring no knowledge of *A*, *P* or *S*. This locates the value in the *observation* of the disaggregated state rather than in the solution of the control problem, which is the paper’s most transferable claim, and the one least dependent on the specific calibration.

Against these, we set what the paper does not establish. It does not estimate K from data; the coupling entries are stipulated, and the Monte Carlo analysis establishes robustness within a neighborhood of that stipulation rather than against structurally different specifications. It does not test any prediction against observation. And its policy conclusions are derived within a linear, time-invariant, single-equilibrium model, which is precisely the class in which crises are least well represented (Section 10).

## 10. Limitations and Ethical Considerations

### 10.1. Modelling Limitations

The present framework makes several simplifying assumptions.

*Linearity.* Equation (10) is linear in x, which is appropriate for small deviations from equilibrium but loses validity during deep crises or structural breaks. Nonlinear extensions—for example, replacing K with a state-dependent operator K(x)—are a natural direction for future work.

*Stationarity of A.* We assume *A* is time-invariant, which by Equation (9) also requires friction to be constant over the horizon. In practice, the coupling tensor K evolves endogenously (e.g., during deleveraging, cross-sector spillovers intensify), and the international evidence indicates that integration raises comovement precisely during crises [21]. A time-varying A(t) would require tracking the spectral abscissa along the trajectory, complicating the stability analysis and placing it outside Proposition 1.

*Heuristic calibration.* The coupling K, the friction rates Γ, and the shock profile of Equation (31) are heuristically specified to represent stylized 2007–2009 transmission channels; the scenario is 2008-*inspired*, not empirically calibrated. Accordingly, quantitative statements in this paper (peak drops, recovery times, the structural lag) are properties of the stylized model, not empirical estimates. The Monte Carlo analysis of Section 8.4 mitigates, but does not remove, this limitation: it establishes robustness of the ordinal conclusions within a neighborhood of the chosen calibration, not against structurally different specifications of K. The empirical route is set out in Section 3.8.

*Low-rank structure.* The parsimony argument of Section 4.3(ii) is conditional on the coupling operator being well approximated at low rank. We do not establish this, and the 3×3 model is too small for its CP rank to bear on the realistic case; whether empirical coupling operators admit accurate low-rank approximations is an open question to be settled with the methods of [31,32].

*Equilibrium uniqueness.* We assume a unique, fixed $M^{*}$. Real economies may have multiple equilibria (stagnation traps, financial acceleration), which would require a bifurcation analysis beyond the linear framework.

### 10.2. Ethical Considerations

A framework that motivates algorithmic allocation of public resources at sector–agent resolution raises governance questions that are not peripheral to it. We address three.

#### 10.2.1. Data Infrastructure and the Surveillance Question

The targeted rule of Section 8.2 conditions on the current deficit of each channel, and therefore requires capital flows resolved by sector and agent class at policy frequency. In the European context, this maps onto supervisory credit registers (AnaCredit; in Portugal the Central de Responsabilidades de Crédito) combined with national input–output accounts. It is worth being precise about what resolution is actually needed: the rule consumes *cell-level aggregates*—nine numbers in the toy model, of order $10^{3}$ for a realistic sectoral and institutional partition—and not transaction-level records. This distinction matters, because it places the requirement within reach of established disclosure-control practice: the necessary statistics can in principle be produced under differential privacy, or through secure multi-party computation across the institutions that already hold the underlying data, without constituting a new centralized transaction database [44]. We do not want to overstate this. The aggregation has to happen somewhere, and whichever institution performs it holds granular data; the honest statement is that the framework does not require a surveillance infrastructure that does not already exist, but neither does it dissolve the risks of the one that does.

#### 10.2.2. Concrete Governance Mechanisms

Appeals to participatory design, impact auditing and human override are empty unless specified for the instrument at hand. For this framework we mean the following. *Participatory design* means deliberation, before deployment, over the reference equilibrium $M^{*}$ and the deficit weights, since these encode the distributive target and are not technical parameters. *Impact auditing* means mandatory publication, after each deployment, of the realized allocation disaggregated by cell together with an assessment of its distributional incidence—which the tensor formulation makes straightforward, since the allocation is already indexed by agent class. *Human override* means a statutory requirement that the deficit-weighted allocation function as a recommendation to a fiscal authority rather than as an executable rule, with published reasons where it is departed from [45,46]. The general point is that the granularity which creates the governance risk is also what makes the incidence of the policy legible; the safeguards should exploit that rather than merely deprecate it.

#### 10.2.3. The Distributive Status of the Reference Equilibrium

The most serious objection to the framework is internal to it. The targeted rule restores the economy to $M^{*}$; if $M^{*}$ embeds an unequal distribution of credit across agent classes—and any equilibrium estimated from historical data will—then deficit-weighted stimulus restores that inequality, and restores it *faster* than an untargeted policy would. Efficiency at returning to an unjust allocation is not a neutral property [47,48,49]. Two observations follow. First, this is a feature of any equilibrium-referenced stabilization rule, including conventional ones, and not a defect peculiar to tensor methods; what is peculiar here is that the incidence becomes visible rather than remaining implicit in an aggregate. Second, the framework admits an alternative: targeting a normative reference $M^{\mathrm{ref}}\neq M^{*}$ chosen by deliberation rather than inherited from the pre-crisis allocation. We note, however, that this converts a stabilization instrument into a redistributive one. That is a political decision and not a modeling choice, and we do not think it can be made on the authority of a model.

## 11. Conclusions

### 11.1. Summary of Findings

We have developed a tensor-based framework for monetary dynamics that formalizes the multidimensional structure of economic capital flows, quantifies the information destroyed by scalar aggregation, assembles from standard Lyapunov theory a stability and convergence framework valid for non-normal coupling, and illustrates the framework through a stylized model.

The principal results are:

1. Within standard Lyapunov theory, the monetary tensor $M_{ij}\left(t\right)$ governed by Equation (10) is globally asymptotically stable whenever α(A)<0, with guaranteed decay rate $1/\lambda_{\max}\left(P\right)$ for V (in the present calibration 0.055 per quarter) and asymptotic rate 2|α(A)| (0.060 per quarter); for the non-normal *A* generated by asymmetric coupling, transient contagion overshoot precedes mean reversion.
2. Cross-sector contagion ($K_{ijkl}\neq 0$) produces a rich propagation pattern under financial shocks: in the stylized calibration, the scalar aggregate contracts by 8.6% while the epicenter contracts by 18.9%—roughly a factor of two of heterogeneity hidden by aggregation, a ratio that exceeds one in 100% of the perturbed ensemble. The information-theoretic diagnostics show further that the aggregate signal normalizes before the sectoral structure does, as  measured by $D_{\mathrm{KL}}\left(p\left(t\right)\;∥\;p^{*}\right)$; the lag is positive in 96.6% of draws, though its magnitude varies widely across calibrations. This masking is not an artifact of the particular scalar chosen: the Frobenius norm (10.1% peak drop) and the leading singular value (8.6%) understate the epicenter’s 18.9% contraction by comparable margins to the sum (Section 5), so the finding is a property of aggregation itself rather than of the summation rule.
3. Under a symmetric exit rule and in the stylized calibration, deficit-targeted fiscal stimulus restores equilibrium in 12.8 quarters against 45.1 for share-weighted stimulus at the same rate and 40.7 with no policy—72% faster than uniform under identical termination, an ordering that holds in 100% of the perturbed ensemble. The advantage is not bought with a larger budget: the targeted rule spends 1.65 against the uniform rule’s 2.00, and  cannot be made to spend much more (it saturates at 1.70), since raising its rate closes the deficits sooner and shortens the active period. Against the exact LQR benchmark, the targeted heuristic attains integrated disequilibrium only 18% above the Pareto frontier at equal control effort, while the uniform heuristic is 2.34 times the frontier.

### 11.2. Theoretical Implications

Three implications follow for the modeling of monetary aggregates. First, the map from the disaggregated state to a scalar aggregate is a coarse-graining whose loss is measurable, not merely arguable: the KL divergence of the normalized flow distribution supplies the measure, and the null model of Section 5 shows that a purely aggregate disturbance registers exactly zero on it, so that the divergence isolates the compositional component by construction. Second, the lost component is not incidental to policy but constitutive of it: the comparison against the LQR frontier in Section 8.5 shows that most of the attainable stabilization gain is available to a rule that merely *observes* the disaggregated state, without solving the control problem. Third, the Lyapunov function offers a disequilibrium indicator that is dynamically consistent with the model’s own recovery geometry—it decreases monotonically in the unforced regime—which conventional gap measures are not guaranteed to be. Whether that property survives in a nonlinear or time-varying specification is open, and we regard it as the most important theoretical question the framework leaves unanswered.

### 11.3. Practical Implications and Policy Recommendations

We state four recommendations, each with the conditions under which it holds. All are derived from a stylized model and require empirical validation before operational use; we set them out concretely so that they are testable rather than merely suggestive.

1. **Publish a structural recovery indicator alongside the aggregate.** Statistical agencies and central banks already collect the sectoral data from which a normalized flow distribution can be constructed. Publishing $D_{\mathrm{KL}}\left(p\left(t\right)∥p^{*}\right)$ and the associated reallocation share δ(t) alongside aggregate credit series would make compositional distress visible at no additional collection cost. The value of doing so is greatest where sectoral heterogeneity is greatest—which is precisely where aggregate series are least informative.
2. **Condition stimulus on deficits rather than on shares.** Where cell-level deficit information is available, allocating in proportion to $\max(-x_{ij},\;0)$ rather than to equilibrium shares dominates in every draw of our ensemble under a common termination rule. The recommendation is conditional on the availability of that information; where it is absent, the relevant comparison is not targeted versus uniform but uniform versus nothing.
3. **Specify an exit rule explicitly.** The secondary experiment of Section 8.3 shows a non-terminating share-weighted rule performing worse than inaction in this calibration, because it continues to inject into channels that have already overshot. A deficit-weighted rule self-terminates by construction; a share-weighted one does not, and must be given a termination condition by design rather than by discretion.
4. **Treat the reference equilibrium as a policy choice.** Since any equilibrium-referenced rule restores the economy to $M^{*}$, the selection of $M^{*}$ is a distributive decision. It should be made deliberately and transparently rather than inherited by default from the pre-crisis allocation (Section 10.2).

### 11.4. Future Work

Future work should address: (i) empirical calibration of K from administrative micro-data following the protocol of Section 3.8; (ii) nonlinear extensions admitting multiple equilibria and state-dependent coupling; (iii) a cross-country extension in which a fourth tensor mode indexes economies and K acquires a country-pair block structure, connecting the framework to the international transmission literature of Section 2.2; (iv) integration with real-time data streams for dynamic policy adjustment; and (v) the governance framework required for responsible algorithmic policy design.

## Figures and Tables

**Table 1:** Operational definition of reported variables. Grid: uniform, 0.05 quarters. All are computed on the deviation trajectory x(t) or on the flow matrix $M\left(t\right)=M^{*}+x\left(t\right)$.

Variable	Definition	Units	Reported in
Peak drop, sector *i*	$\max_{t}\left[1-\sum_{j}M_{ij}\left(t\right)/\sum_{j}M_{ij}^{*}\right]$	%	Section 7 and Section 8.4
Aggregate drop	$\max_{t}\left[1-S\left(t\right)/S^{*}\right]$	%	Section 7
Epicenter/aggregate ratio	ratio of the two preceding quantities	—	Section 8.4
Recovery time $\tau_{r}$	first *t* with $V\left(t\right)<0.05\max_{s}V\left(s\right)$ and V below that level for all later times	q	Section 8.2, Section 8.3, Section 8.4 and Section 8.5
Entropy H(t)	Equation (15)	nats	Section 7.4
Structural displacement $D_{\mathrm{KL}}\left(t\right)$	Equation (16)	nats	Section 7.4
Reallocation share δ(t)	$\frac{1}{2}\sum_{ij}\left|p_{ij}\left(t\right)-p_{ij}^{*}\right|$	% of S	Section 7.4
Mutual information I(S;A)(t)	Equation (18)	nats	Section 7.4
Structural lag	$t_{\mathrm{rec}}\left(D_{\mathrm{KL}}\right)-t_{\mathrm{rec}}\left(|\Delta S|\right)$, each at its own 5% threshold	q	Section 7.4
Integrated disequilibrium $J_{V}$	$\int_{0}^{T}V\left(t\right)\;dt$	—	Section 8.5
Control effort $J_{u}$	$\int_{0}^{T}{∥u\left(t\right)∥}^{2}\;dt$	—	Section 8.5
Gross expenditure	$\int_{0}^{T}\sum_{ij}\left|u_{ij}\left(t\right)\right|\;dt$	norm. units	Section 8.5
Net expenditure	$\int_{0}^{T}\sum_{ij}u_{ij}\left(t\right)\;dt$	norm. units	Section 8.5

**Table 2:** Summary of notation.

Symbol	Meaning	First Appearance
M	monetary tensor, $N_{S}\times N_{A}\times T$	Section 4
M(t)	monetary matrix (mode-3 slice)	Equation (5)
$M^{*}$	equilibrium flow matrix	Equation (24)
$x_{ij}$, x	deviation from equilibrium; its vectorization	Equation (10)
K, $K_{\mathrm{flat}}$	coupling tensor; its matricization	Equations (8) and (11)
$\Gamma_{ij}$	channel friction rate (constant in time)	Equation (9)
*A*	system matrix	Equation (12)
α(A)	spectral abscissa	Proposition 1
*P*	Lyapunov matrix	Equation (19)
V	Lyapunov function $x^{⊤}Px$	Proposition 1
κ(P)	condition number of *P* (reserved for this use)	Equation (22)
S(t)	scalar aggregate $\sum_{ij}M_{ij}\left(t\right)$	Section 5
$p_{ij}$, $p_{i\cdot }$, $p_{\cdot j}$	normalized flow distribution and its marginals	Equations (14) and (18)
*H*, $D_{\mathrm{KL}}$, I(S;A), δ	entropy, divergence, mutual information, reallocation share	Section 5
*B*, *S*, ρ	policy transmission matrix, Riccati solution, budget parameter	Section 8.1
λ, $T_{\mathrm{pol}}$, $t_{0}$	policy rate, window length, deployment time	Section 8.2
$\tau_{r}$	recovery time	Table 1

**Table 3:** Non-zero entries of the coupling tensor. Following the target-first convention of Equation (8), the entry $K_{\left(ij)(kl\right)}$ has target (ij) and source (kl); the columns below are labeled accordingly, so that no inference from index ordering is required. Negative entries represent contagion; positive entries represent complementarity or stabilization. The final row is a self-coupling that lowers the effective friction of the Services–Government channel from $\Gamma_{\mathrm{SG}}=0.05$ to 0.03 per quarter; this channel is the slowest mode of the system and sets the spectral abscissa α(A)=−0.030. The mechanisms are described in the text below.

Source (kl)	Target (ij)	$K_{\left(\mathrm{ij})(\mathrm{kl}\right)}$	Mechanism
F/Fi	M/Fi	−0.08	Credit crunch
F/Fi	M/H	−0.05	Consumer demand
F/H	M/H	−0.04	Household credit
F/G	M/G	−0.02	Fiscal contraction
F/Fi	S/Fi	−0.07	Corporate lending
F/H	S/H	−0.04	Consumer credit
M/Fi	S/Fi	+0.03	Supply chain
M/H	S/H	+0.02	Demand spillover
M/H	F/H	−0.02	Feedback to Finance
S/G	M/G	+0.02	Fiscal stabilizer
S/G	S/G	+0.02	Fiscal stabilizer (self)

**Table 4:** Entropic diagnostics under stochastic forcing, $\sigma_{ij}=\sigma M_{ij}^{*}$, Euler–Maruyama with Δt=0.01 q over 400 paths. The floor is the ensemble-mean divergence for t>60 q, where the shock has decayed and the process is at its stationary law. The deterministic (σ=0) reference values are $D_{\mathrm{KL}}^{\max}=8.5\times 10^{-3}$ nats and a structural lag of 21.0 quarters.

σ	$\mathrm{tr}\;\Sigma_{\infty }$	KL Floor	Peak $E[D_{\mathrm{KL}}]$	Peak/Floor	lag >0
0.005	$8.6\times 10^{-3}$	$8.0\times 10^{-5}$	$8.6\times 10^{-3}$	109	100.0%
0.010	$3.4\times 10^{-2}$	$3.0\times 10^{-4}$	$8.8\times 10^{-3}$	29.1	100.0%
0.020	$1.4\times 10^{-1}$	$1.2\times 10^{-3}$	$9.5\times 10^{-3}$	7.9	99.2%

**Table 5:** Monte Carlo sensitivity analysis over N=1000 perturbed economies (K entries ±50%, Γ±30%, shock amplitudes ±30%, decays ±20%; all draws stable). Medians and 5th–95th percentile ranges; recovery times right-censored at 120 quarters (binding for 11.6% of draws under the non-terminating uniform policy and 2.0% under the exit-rule uniform policy; never binding for the no-policy and targeted scenarios). Note the width of the structural-lag range, discussed in the text.

Quantity	Baseline Value	Median	[5th, 95th] pct
$\tau_{r}$, no policy (q)	40.7	41.5	[30.4, 50.4]
$\tau_{r}$, uniform, non-terminating (q) *^a^*	69.9	68.8	[49.5, ≥120]
$\tau_{r}$, targeted, non-terminating (q)	13.7	15.0	[12.8, 20.0]
$\tau_{r}$, uniform, exit rule (q)	45.1	47.4	[39.2, 82.2]
$\tau_{r}$, targeted, exit rule (q)	12.8	13.7	[12.2, 35.4]
Finance peak drop (%)	18.9	19.2	[13.1, 26.6]
Manufacturing peak drop (%)	5.8	5.9	[3.7, 8.6]
Services peak drop (%)	3.9	3.9	[2.2, 6.5]
Aggregate peak drop (%)	8.6	8.7	[7.3, 10.5]
Epicenter/aggregate ratio	2.2	2.2	[1.6, 2.9]
Structural lag $t_{\mathrm{rec}}\left(D_{\mathrm{KL}}\right)-t_{\mathrm{rec}}\left(|\Delta S|\right)$ (q)	21.0	22.1	[1.8, 34.7]

*^a^* Right-censored in 11.6% of draws: the 95th percentile is a lower bound.

## Data Availability

All simulation code, numerical data, and scripts required to reproduce the figures and numerical results reported in this article are publicly available in the GitHub repository *Econophysics*: https://github.com/mjgpinheiro/Econophysics (accessed on 8 July 2026). The main notebook Money_Tensor_crisis_model.ipynb reproduces the baseline simulations, while the folder sensitivity_analysis/ contains the scripts, pre-computed data, and figures for the Monte Carlo sensitivity analysis and the LQR benchmark (Section 8.4 and Section 8.5). The folder revision/ contains the scripts added in the revision: the stochastic-forcing experiment of Section 7.5, the alternative-scalarization and null-model checks of Section 5, the CP-rank residual computation of Section 4.3, the expenditure sweep and maximum-attainable-expenditure policy variant of Section 8.2, and the fixed-step self-convergence and smooth-regularization checks of Section 3.5. All random seeds are fixed and recorded.

## Supplementary Materials

The following supporting information can be downloaded at: https://www.mdpi.com/article/10.3390/e28080915/s1: Figure S1: complete 3×3 array of the monetary tensor $M_{ij}\left(t\right)$ under the stylized shock, all nine sector–agent channels on a common vertical scale; Table S1: residual norms of the numerical solutions of the algebraic Lyapunov and Riccati equations; Table S2: relative CP-fit residual of the simulated trajectory tensor as a function of the rank *R*; Figure S2: trajectories of the alternative scalarizations ${∥M\left(t\right)∥}_{F}$ and $\sigma_{1}\left(M\left(t\right)\right)$ alongside S(t), together with the proportional-shock null model.
