# Supplementary material for: Information Loss in Scalar Monetary Aggregation: A Tensorial Langevin Framework for Financial Shock Propagation and Policy Targeting
Source: Entropy (Basel). 2026 Aug 14;28(8):915. doi: 10.3390/e28080915 (PMC13511854; doi:10.3390/e28080915)
Supplement: Supplementary file 1 [file entropy-28-00915-s001.zip › entropy-4458654-supplementary.pdf]

Supplementary Materials

Information Loss in Scalar Monetary Aggregation: A Tensorial Langevin Framework for Financial Shock Propagation and Policy Targeting

M. Rodrigo Pinheiro   Mario J. Pinheiro

Figure S1: Complete sector–agent trajectories

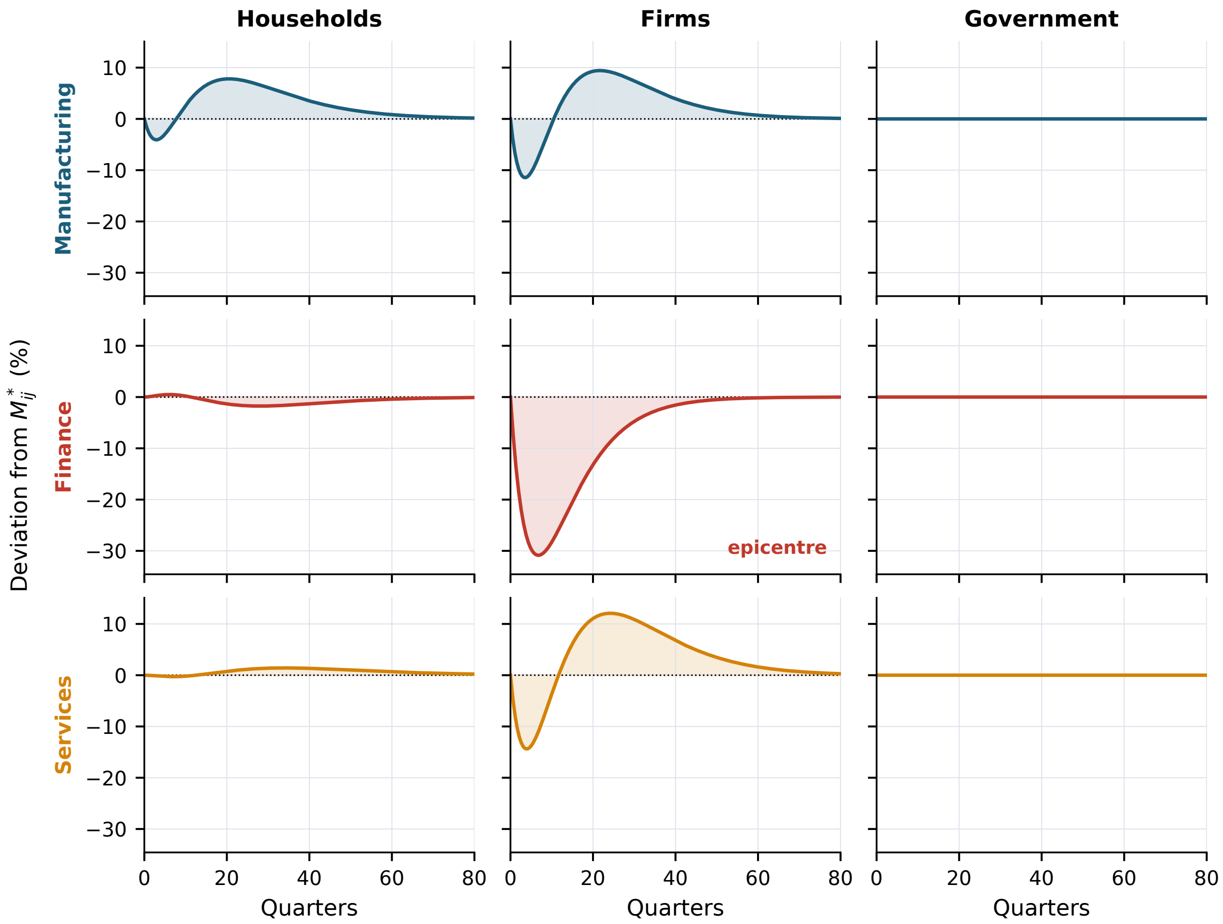

Figure S1. Complete 3×3 array of the monetary tensor $\mathcal{M}_{ij}(t)$ under the stylized shock, showing all nine sector–agent channels on a common vertical scale. Curves are percentage deviations from the corresponding equilibrium value M_{ij}^*. The Finance–Firms channel is the shock epicenter.

Table S1: Algebraic-equation residuals

The numerical solutions of the Lyapunov and Riccati equations were checked by direct substitution. For the Lyapunov equation the residual is

$$R_L = A^\top P + PA + I,$$

and for the LQR benchmark at $\rho = 128$ and $B = I$ the Riccati residual is

$$R_R = A^\top S + SA - \rho^{-1} S^2 + P.$$

Table S1. Residual norms for the algebraic Lyapunov and Riccati solutions.

Equation	$\|R\|_F$	$\max_{ij} \|R_{ij}\|$	Relative Frobenius residual
Lyapunov	5.24×10^{-16}	4.44×10^{-16}	1.75×10^{-16}
Riccati ($\rho = 128$)	6.26×10^{-14}	2.31×10^{-14}	2.55×10^{-15}

For the relative residual, the denominator is $\|I\|_F$ for the Lyapunov equation and $\|P\|_F$ for the Riccati equation. The values are at floating-point roundoff level for the Schur-method solutions used in the manuscript.

Table S2: CP-fit residual versus rank

The simulated no-policy trajectory was sampled at 200 equally spaced times and fitted by CP alternating least squares. To reproduce the values quoted in the manuscript, five deterministic starts (random seeds 0–4) were run for each rank, with at most 800 ALS iterations and a convergence tolerance of 10^{-12}; the smallest residual among the five starts is reported.

Table S2. Relative CP-fit residual $\|\mathcal{M} - \widehat{\mathcal{M}}\|_F / \|\mathcal{M}\|_F$ for the simulated $3 \times 3 \times T$ trajectory tensor.

CP rank R	Relative residual
1	2.728×10^{-1}
2	3.547×10^{-2}
3	7.392×10^{-3}

As emphasized in the manuscript, this small $3 \times 3 \times T$ exercise is a numerical diagnostic only and does not establish low-rank structure for a realistically larger empirical tensor.

Figure S2: Alternative scalarizations and proportional-shock null model

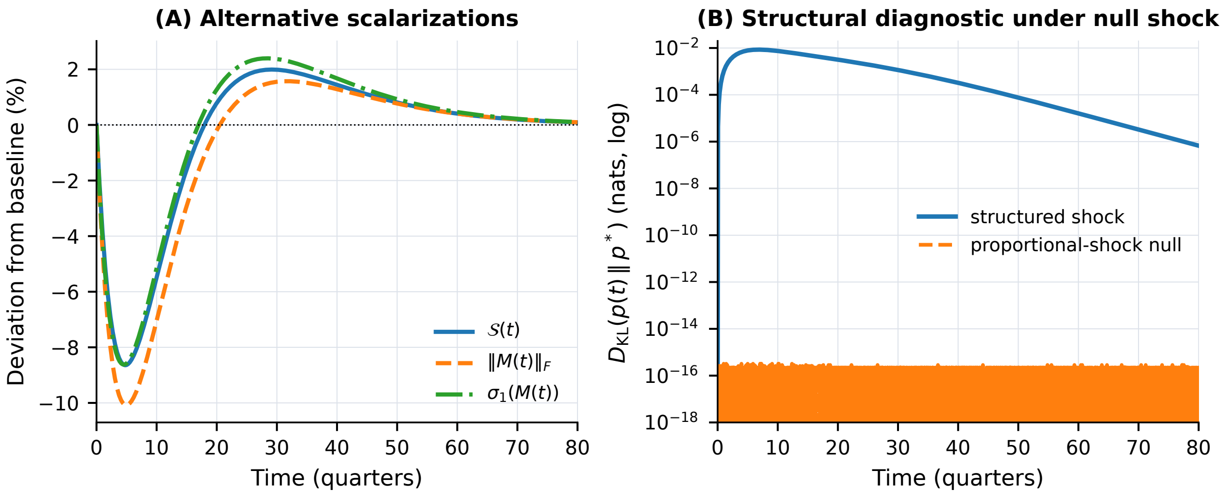

Figure S2. (A) Alternative scalarizations of the same simulated monetary state: the total $\mathcal{S}(t)$, Frobenius norm $\|M(t)\|_F$, and leading singular value $\sigma_1(M(t))$, each shown as a percentage deviation from its baseline. Their peak drops are respectively 8.64%, 10.08%, and 8.62%, all below the Finance-sector peak drop of 18.88%. **(B)** Structural KL diagnostic for the full heterogeneous shock and for a proportional-shock null model. Under the proportional null, D_{KL} remains at machine precision (maximum 3.23×10^{-16} nats), confirming that the structural diagnostic responds to compositional displacement rather than to a purely aggregate rescaling.
